# HPV E7 inhibits cell pyroptosis by promoting TRIM21-mediated degradation and ubiquitination of the IFI16 inflammasome

**DOI:** 10.7150/ijbs.50074

**Published:** 2020-09-13

**Authors:** Yinjing Song, Xia Wu, Yaohan Xu, Jiang Zhu, Jiaying Li, Ziqi Zou, Luxia Chen, Boya Zhang, Chunting Hua, Han Rui, Qiaoli Zheng, Qiang Zhou, Qingqing Wang, Hao Cheng

**Affiliations:** 1 Department of Dermatology and Venereology, Sir Run Run Shaw Hospital, Zhejiang University School of Medicine, Hangzhou 310016, Zhejiang, PR China.; 2 Institute of Immunology, Zhejiang University School of Medicine, Hangzhou 310058, China.

**Keywords:** Human papillomavirus E7, pyroptosis, ubiquitination, inflammasome

## Abstract

Human papillomavirus (HPV) is a DNA virus that causes sexually transmitted infections. The HPV oncoprotein E7 plays a critical role in the regulation of host immunity to promote the immune escape of HPV and the occurrence of cervical cancer or genital warts. Pyroptosis, a highly inflammatory form of programmed cell death, can be induced by inflammasomes and acts as a defense against pathogenic infection. However, whether HPV E7 can regulate cell pyroptosis to evade immune surveillance has not been determined. In this study, we found that HPV E7 could inhibit cell pyroptosis induced by transfection with dsDNA. The activation of the inflammasome, and the production of IL-18 and IL-1β were also restrained by HPV E7. Mass spectrometry and immunoprecipitation showed that HPV E7 interacted with IFI16 and TRIM21. We also discovered that HPV E7 recruited the E3 ligase TRIM21 to ubiquitinate and degrade the IFI16 inflammasome, leading to the inhibition of cell pyroptosis and self-escape from immune surveillance. Thus, our study reveals an important immune escape mechanism in HPV infection and may provide targets for the development of a novel immunotherapeutic strategy to effectively restore antiviral immunity.

## Introduction

Human papillomavirus (HPV) is a small double-stranded DNA virus that infects skin or mucosal cells and causes the development of genital warts or cancers 1. Depending on the oncogenic potential cancers, HPV types are classified into low-risk types such as HPV-6 and HPV-11 which are found mainly in genital warts, and high-risk types, such as HPV-16 and HPV-18 which are frequently associated with cervical cancer 2. The induction of immune evasion by HPV is one of the most important mechanisms in persistent HPV infection and is responsible for HPV-associated disease progression 3. Accumulating evidence shows that the both low-risk and high-risk HPV E7 plays an important role in immune evasion through various mechanisms 4-6. For example, both low-risk and high-risk HPV E7 proteins inhibit the expression of IRF1 by recruiting HDAC to promoters that contain IRF1 response elements 7. The production of IL-18 binding protein (IL-18BP) which can prevent the deleterious effects of excessive IL-18 secretion is increased in primary human keratinocytes expressing either high-or low-risk HPVE7 8. HPV E7 also restrain the expression of interferons (IFNs), proinflammatory cytokines, chemokines, TLRs and MHC-I by deregulating host DNA methylation and histone modification 9-12. Moreover, HPV E7 can locate in the cytoplasm to interrupt the cGAS-STING and IFN signaling pathways by interacting with STING, IRF9 or IRF1 13-15. Despite these findings, the break in host immune surveillance induced by HPV E7 is far from fully understood, which largely restricts the efficacy of immunotherapy against HPV.

Host cell death, including apoptosis, necroptosis, and pyroptosis, is critical for host defenses against infection and the clearing of intracellular pathogens 16, 17. Pyroptosis is a recently identified host cell death pathway that occurs most frequently upon infection with intracellular pathogens 18, 19. It has recently been reported that pyroptosis is mediated by gasdermin D (GSDMD), which is cleaved by the inflammatory caspase-1 or caspase-11 and forms pores on membranes, resulting in the extracellular release of IL-1β and-18, cell swelling and pyrolysis 20, 21. Nucleotide-binding oligomerization domain-like receptors (NLRs) and absent in melanoma 2 (AIM2)-like receptors are able to form multimeric inflammasome complexes and activate inflammatory caspases which initiate cell pyroptosis during pathogen infection 22. Interferon-gamma inducible protein 16 (IFI16) is a member of the PYHIN family, and in addition to being able to promote cGAS-STING-dependent interferon-β (IFN-β) production, IFI16 can also assemble the inflammasome and initiate pyroptosis 23, 24. For example, IFI16 detects viral DNA and initiates the assembly of inflammasome in both the cytoplasm and nucleus upon infection with DNA viruses such as Kaposi's sarcoma-associated herpesvirus (KSHV), Epstein-Barr virus (EBV) or herpes simplex virus (HSV-1) 25-28. Moreover, human immunodeficiency virus 29 DNA can be sensed by IFI16 and induce the activation of IFI16 inflammasome in the cytoplasm, which mediates the pyroptosis of CD4^+^ T cells during abortive HIV-1 infection 30. Therefore, the IFI16 inflammasome plays an important role in the initiation of pyroptosis during viral infection. However, whether HPV infection or HPV oncoproteins affect IFI16 inflammasome-mediated pyroptosis is unclear.

Here, we found that HPV E7 could inhibit cell pyroptosis induced by the IFI16 inflammasome. Mass spectrometry and immunoprecipitation showed that HPV E7 interacted with IFI16 and TRIM21. Furthermore, we found that HPV E7 promoted the K33-linked ubiquitination and degradation of IFI16 mediated by the E3 ligase TRIM21. Our data demonstrated that HPV E7 inhibited cell pyroptosis by promoting TRIM21-mediated degradation and K33-linked ubiquitination of the IFI16 inflammasome, thereby facilitating HPV immune escape.

## Materials and Methods

### Plasmids, antibodies, and reagents

Recombinant vectors encoding human IFI16 (NM_001364867.2), TRIM21 (NM_003141.4), and HPV 18E7 (NC_001357.1) were generated by PCR-based amplification of HeLa cell cDNA, followed by subcloning into the pcDNA3.1 eukaryotic expression vector or pEGFP-C1 vector. Complementary DNA (cDNA) for HPV 11E7 (GenBank: CCC55776.1) was amplified from condyloma acuminate tissues, and HPV 18E7 cDNAs were inserted into the pHAGE-fEF1a-IRES-ZsGreen lentiviral vector with an N-terminal Flag tag for lentiviral packaging. All constructs were confirmed by DNA sequencing. 18E7 siRNA was purchased from GenePharma.

Antibodies against GSDMD (96458s, 1:3000), caspase-1 (3866T, 1:3000), cleaved caspase-1 (4199T, 1:3000), and GAPDH (5174, 1:5000) were purchased from Cell Signaling Technology. Antibodies specific for IFI16 (sc-8023, 1:1000), TRIM21 (sc25351, 1:1000), ubiquitin (sc-471120, 1:1000), and GFP (sc-390394) were from Santa Cruz, Inc. Antibodies against Flag (M20008M), Myc (M20002M), mouse IgG (B30010M), and rabbit IgG (B30011S) were obtained from Abmart. Poly(dA:dT) (P0883), MG132 (M8699), CHX (C4859) and anti-FlagM2 magnetic beads (M8823) were purchased from Sigma-Aldrich. ELISA kits for human IL-18 and IL-1β were purchased from eBioscience.

### Cell culture and transfection

The HeLa cervical cancer cell line (ATCC no. CCL2) and 293T cells (ATCC no. CRL-3216) were purchased from the American Type Culture Collection (ATCC, Manassas, VA, USA) and maintained in Dulbecco's modified Eagle's medium (DMEM) containing 10% fetal bovine serum. For transient transfection, HeLa and 293T cells were transfected with plasmids using jetPRIME (PolyPlus-Transfection, 114-15). To develop stable expression cell lines, HaCaT or HeLa cells were transfected with lentivirus for 48 hr and sorted by flow cytometry (BD Biosciences FACSAria II).

### Generation of knockout cell lines by CRISPR-Cas9

pGE-4 (pU6gRNA1U6gRNA2Cas9puro) CRISPR-Cas9 plasmids were purchased from Gene Pharma. The target sequences used were ATGCTCACAGGCTCCACGAA and CATGTTGGCTAGCTGTCGAT for TRIM21; and CTCAGTACCTTCACTATCAC and GGAATATGATAGTCTCCTAG for IFI16. To construct the knockout cell lines, HeLa cells were transfected with CRISPR-Cas9 and pEGFP-C1 vectors for 48 hr and sorted into 96-well plates for single-cell sorting with a FACSAria II cell sorter (BD BioSciences). The candidate knockout clones were verified by western blotting.

### Mass spectrometry

HaCaT cells stably expressing Flag-HPV 11E7 were transfected with poly(dA:dT) for 6 hrs before collection. Cell lysates were immunoprecipitated with antibody against the Flag epitope tag, and mass spectrometry was used to identify Flag-HPV 11E7-interacting proteins with an Orbitrap Elite mass spectrometer (Thermo Fisher).

### Cytokine release assay

IL-18 and IL-β cytokines were detected with ELISA kits according to the manufacturer's protocols.

### Immunoprecipitation and immunoblot analysis

For immunoprecipitation, cells were lysed in NP-40 lysis buffer (Beyotime, P0013F) with protease inhibitor 'cocktail' (Sigma, P8340) and then centrifuged for 15 min at 12,000 ×g. The supernatants were collected and incubated with protein A/G magnetic beads (Thermo Scientific Pierce, 88802) and a specific antibody at 4°C overnight. Immunoprecipitates were washed five times with IP wash buffer and eluted in SDS-PAGE loading buffer. For immunoblot analysis, whole-cell extracts were lysed with cell lysis buffer (Cell Signaling Technology, 9803) supplemented with a protease inhibitor 'cocktail' (Sigma, P8340). Protein concentrations in the extracts were measured by BCA assay (Pierce, 23227) according to the protocol. Equal amounts of extracts were separated by SDS-polyacrylamide gel electrophoresis and then transferred onto a polyvinylidene fluoride membrane (Millipore, IPVH00010). The polyvinylidene fluoride membrane was blocked with 5% dry nonfat milk in Tris-buffered saline (pH 7.4) containing 0.1% Tween20 and probed with the appropriate antibody for immunoblot analysis.

### Flow cytometry

HeLa cells were transfected with poly(dA:dT) for the indicated times, washed twice with cold PBS, and then resuspended in 1×binding buffer containing propidium iodide (BD Biosciences, 556547) for 15 min at RT (25°C) in the dark. The stained cells were analyzed by flow cytometry (FACS).

### Cell viability and cell death

For cell viability assays, HeLa or HaCaT cells were transfected with poly(dA:dT) for the indicated times, and their viability was detected with a CellTiter-Glo Luminescent Cell Viability Assay (Promega, G7570). Cell death was measured with an LDH assay using a CytoTox 96 Non-Radioactive Cytotoxicity assay kit (Promega, G1780).

### Statistical Analysis

All results are expressed as the mean ± SEM of at least three independent experiments. Statistical significance was evaluated by Student's *t*-test. *P* values less than 0.05 indicated statistical significance.

### Data availability

All relevant data are available from the authors upon request.

## Results

### HPV E7 inhibits pyroptosis induced by intracellular dsDNA

**Figure 1:**
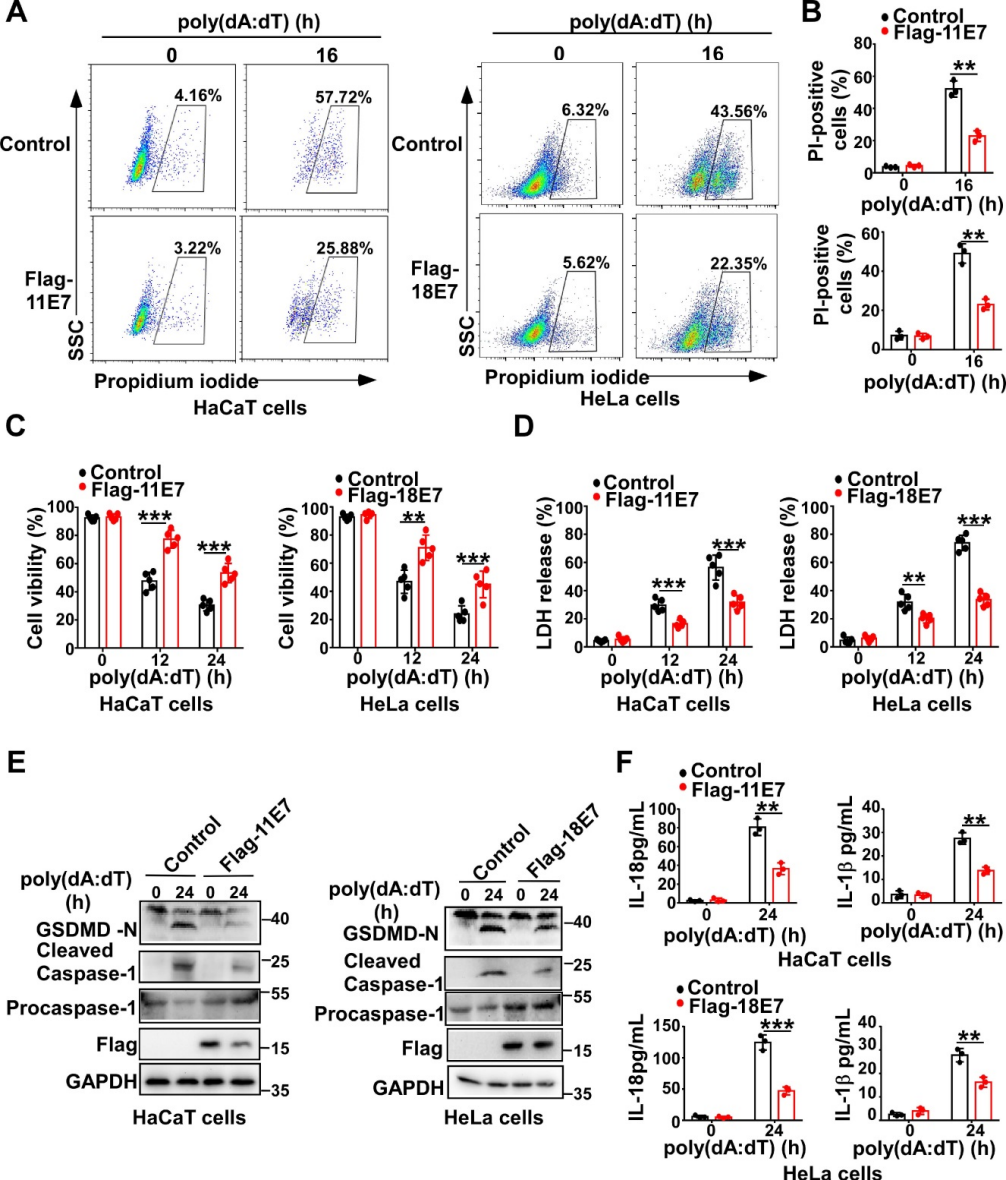
** HPV E7 inhibits pyroptosis induced by intracellular dsDNA.** (A-B) Flow cytometry analysis (A) and statistical analysis (B) of propidium iodide-positive HaCaT cells stably expressing HPV 11E7 or HeLa cells stably expressing HPV 18E7 after transfection with poly(dA:dT) for the indicated times. (C-D) Cell viability assay (C) and LDH assay (D) of HaCaT cells stably expressing HPV 11E7 or HeLa cells stably expressing HPV 18E7 after transfection with poly(dA:dT) for the indicated times. (E) Immunoblot analysis of GSDMD and caspase-1 in the lysates of HaCaT cells stably expressing HPV 11E7 or HeLa cells stably expressing HPV 18E7 after transfection with poly(dA:dT) for the indicated times. (F) ELISA analysis of IL-18 and IL-1β in HaCaT cells stably expressing HPV 11E7 and HeLa cells stably expressing HPV 18E7 after transfection with poly(dA:dT) for the indicated times. Data are presented as mean ± SD of duplicate samples and are representative of at least three independent experiments. P values are determined by two-tailed Student's *t* test. **p < 0.01, ***p < 0.001.

To determine whether HPV E7 can regulate pyroptosis, we constructed and obtained HaCaT cells stably overexpressing low-risk HPV11 early gene E7 or HeLa cells stably overexpressing high-risk HPV18 oncoprotein E7 by infection with the lentivirus expressing Flag-HPV 11E7 or Flag-HPV 18E7, respectively (Fig. S1A). Flow cytometry analysis revealed that fewer HPV E7-overexpressing cells were propidium iodide-positive than control cells in response to transfection with poly(dA:dT) which is a repetitive synthetic double-stranded DNA sequence and recognized by multiple PRRs, such as cGAS, IFI16, DAI, AIM2, DDX41, and LRRFIP1 (Figure 1A-B). Compared to control cells, HPV E7-overexpressing cells displayed higher cell viability, as detected by the CellTiter-Glo luminescent cell viability assay (Figure 1C). Furthermore, the lactate dehydrogenase release assay showed that HPV E7-overexpressing cells released less LDH than control cells in response to poly(dA:dT) (Figure 1D). We also found that overexpression of HPV E7 inhibited the cleavage of GSDMD in HaCaT or HeLa cells in response to transfection with poly(dA:dT) (Figure 1E). As GSDMD is cleaved by inflammatory caspase, we detected the activation of caspase-1 and found that HPV E7-overexpressing cells displayed a lower level of activated caspase-1 than control cells (Fig. 1E). The levels of IL-β and IL-18 were significantly decreased in HPV E7-overexpressing cells transfected with poly(dA:dT) (Figure 1F).Taken together, our results demonstrated that HPV E7 can inhibit cell pyroptosis induced by poly(dA:dT) transfection.

To further confirm the function of HPV E7 in regulating pyroptosis, we silenced 18E7 by shRNA in HeLa cells and transfected with poly(dA:dT). More 18E7 silenced HeLa cells were propidium iodide-positive than control HeLa cells in response to poly(dA:dT) (Figure S1B-1C). More LDH was released from 18E7 silenced HeLa cells than control cells in response to poly(dA:dT) (Figure S1D). However, the cell viability was significantly lower in 18E7 silenced HeLa cells than control HeLa cells after transfection with poly(dA:dT) (Figure S1E). Silence of 18E7 promoted the cleavage of GSDMD and activation of caspase-1 in HeLa cells transfected with poly(dA:dT) (Figure S1F). Furthermore, the production of IL-1β and IL-18 was markedly increased in 18E7 silenced HeLa cells transfected with poly(dA:dT) compared to control HeLa cells transfected with poly(dA:dT) (Figure S1G). Taken together, our results demonstrate that HPVE7 can inhibit cell pyroptosis induced by poly(dA:dT) transfection.

### HPV E7 interacts with IFI16

**Figure 2:**
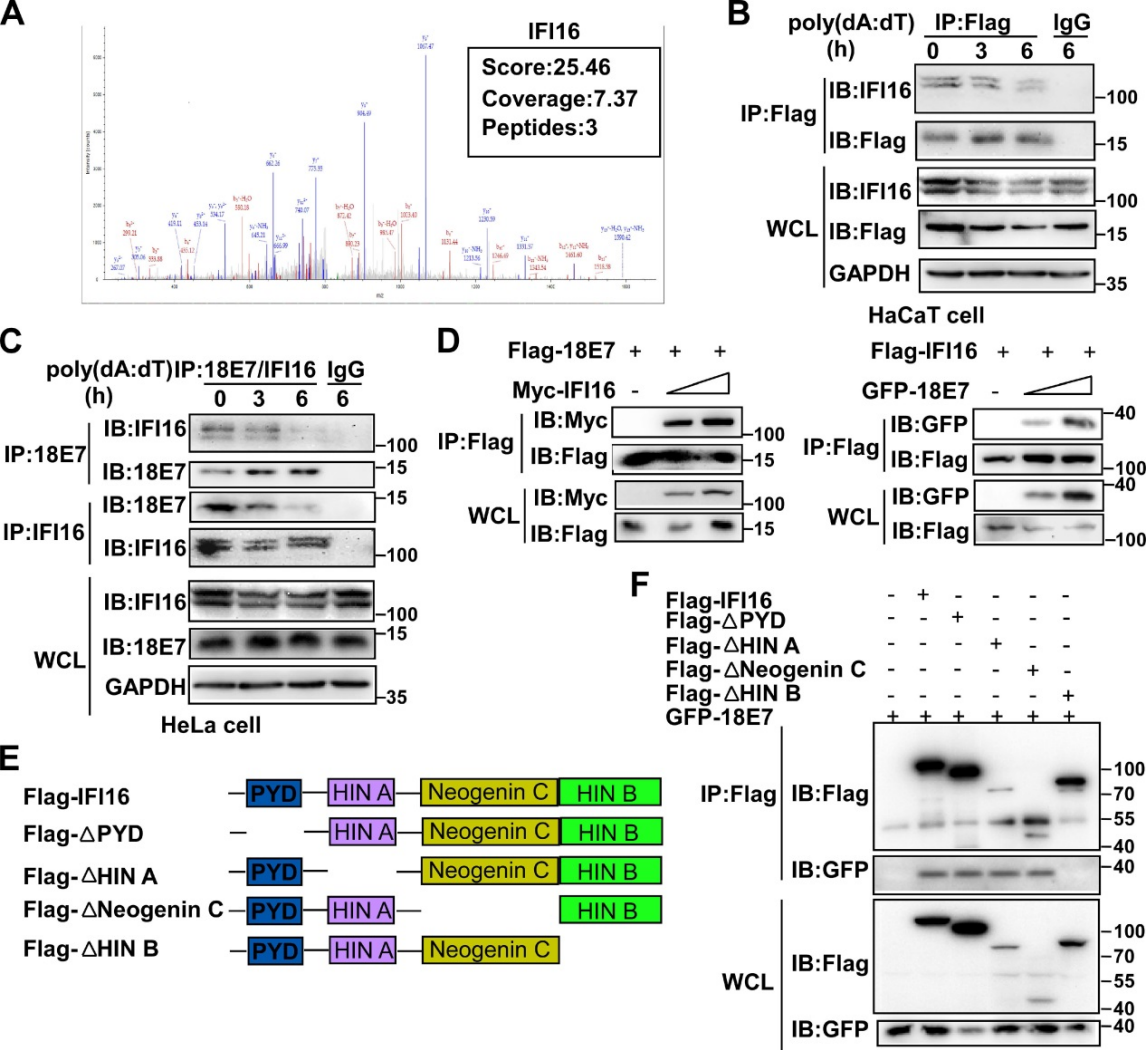
** HPV E7 interacts with IFI16.** (A) Mass spectrum data on IFI16 among HPV 11E7-interacting proteins identified by mass spectrometry. (B) Coimmunoprecipitation and immunoblot analysis of HaCaT cells stably expressing HPV 11E7 after transfection with poly(dA:dT) for the indicated times. (C) Immunoblot analysis of HeLa cells transfected with poly(dA:dT) for the indicated times, followed by immunoprecipitation with IFI16, HPV 18E7 or immunoglobulin G (IgG)-conjugated magnetic beads. (D) Coimmunoprecipitation and immunoblot analysis of 293T cells cotransfected for 36 hr with Flag-HPV 18E7 and Myc-IFI16 or Flag-IFI16 and GFP-HPV 18E7 plasmids followed by immunoprecipitation with anti-FlagM2 beads. (E) A schematic structure and IFI16 derivatives were shown. (F) Immunoblot analysis of 293T cells cotransfected for 36 hr with GFP-HPV 18E7 plus Flag-IFI16 or Flag-IFI16 mutant vectors, followed by immunoprecipitation with anti-FlagM2 beads. Data are representative of at least three independent experiments.

To explore the mechanisms by which HPV E7 inhibited cell pyroptosis, we first detected the mRNA levels of pyroptosis-associated molecules by RNA-seq. There was no obvious difference in the mRNA levels of pyroptosis-associated genes between control HaCaT cells and HPV 11E7-overexpressing HaCaT cells (Figure S2A). Next, we investigated HPV 11E7-interacting proteins by immunoprecipitation (IP) and mass spectrometry. The information for the HPV 11E7-interacting proteins was shown in Supplementary Table 1. Among the HPV 11E7-interacting proteins, we focused on the DNA sensor IFI16, which can assemble the IFI16 inflammasome and initiate cell pyroptosis (Figure 2A). To confirm the interaction between HPV 11E7 and IFI16, we immunoprecipitated the Flag epitope tag from the lysates of poly(dA:dT)-transfected cells overexpressing Flag-HPV 11E7, and immunoblot assay revealed that the interaction between HPV 11E7 and IFI16 was decreased by poly(dA:dT) transfection (Figure 2B). Additionally, the endogenous interaction between HPV 18E7 and IFI16 was reduced in HeLa cells after poly(dA:dT) transfection (Figure 2C). Co-IP and immunoblot assays showed that HPV 18E7 interacted with IFI16 in 293T cells which were transfected with plasmids for HPV 18E7 and IFI16 expression (Figure 2D). IFI16 contains PYD, HINA, HINB, and Neogenin C domains 31. To identify which domain of IFI16 was responsible for the interaction with HPV E7, we constructed the mutant IFI16 plasmids (Figure 2E). Co-IP and western blot assays showed that the HINB domain of IFI16 interacted with HPV 18E7 (Figure 2F). These results strongly indicated that HPV E7 can interact with IFI16.

### HPV E7 promotes the ubiquitin-proteasome-mediated degradation of IFI16

**Figure 3:**
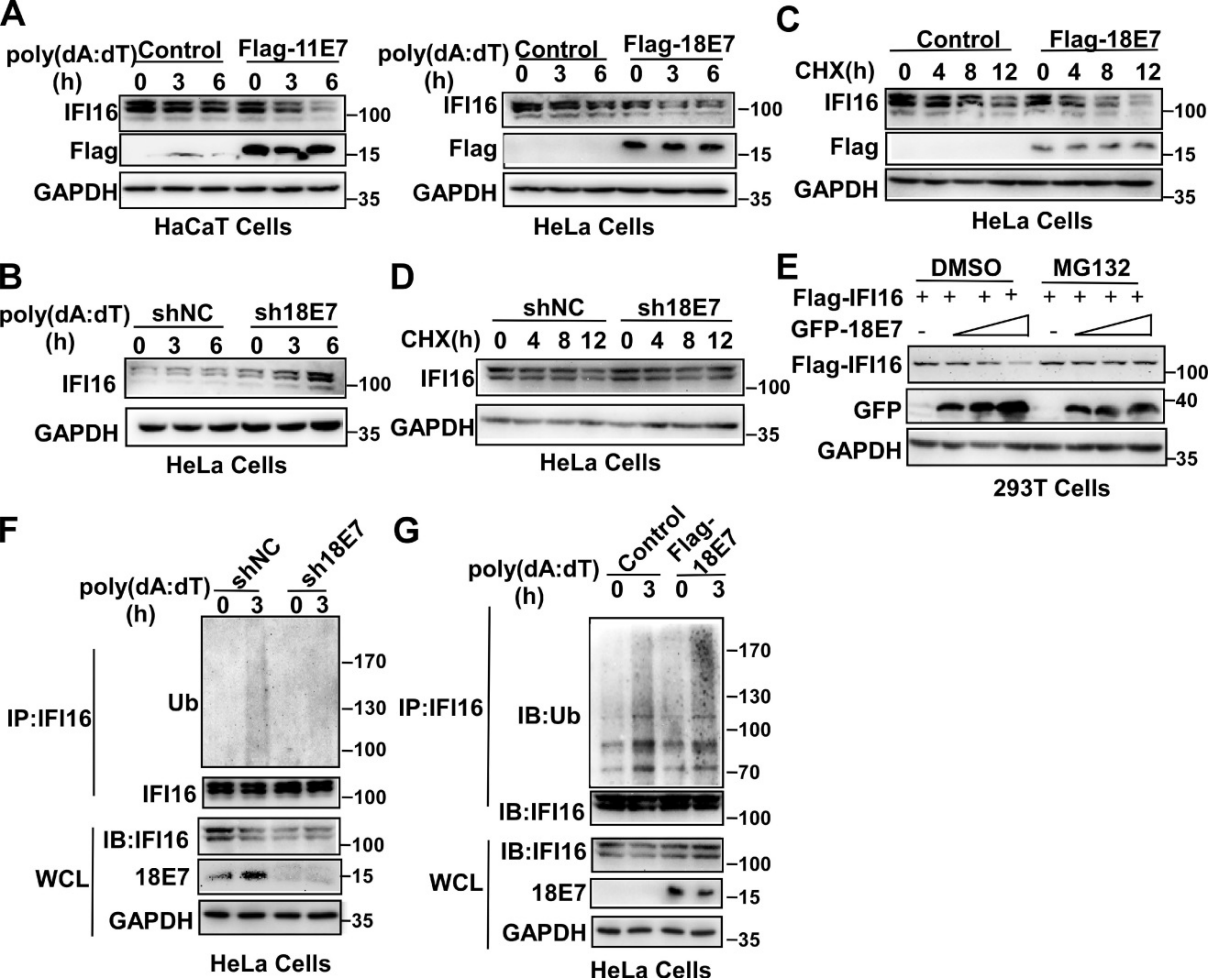
** HPV E7 promotes the ubiquitin-proteasome-mediated degradation of IFI16.** (A) Immunoblot analysis of HaCaT cells stably expressing HPV 11E7 or HeLa cells stably expressing HPV 18E7 transfected with poly(dA:dT) for the indicated times. (B) Immunoblot analysis of the lysates of HeLa cells transfected with shNC or sh18E7 plasmids for 36 hr and transfected with poly(dA:dT) for the indicated times. (C) Immunoblot analysis of control HeLa cells or stably expressing HPV 18E7 HeLa cells treated with CHX (40 μg/ml) for the indicated times after transfection with poly(dA:dT) for 1 hr. (D) Immunoblot analysis of IFI16 in the lysates of control HeLa cells or HPV 18E7-silenced HeLa cells treated with CHX (40 μg/ml) for the indicated times after transfection with poly(dA:dT) for 1 hr. (E) Immunoblot analysis of 293T cells cotransfected with the GFP-HPV 18E7 and Flag-IFI16 vectors with or without MG132 treatment. (F) Immunoblot analysis of ubiquitinated IFI16 in control HeLa cells or HPV 18E7-silenced HeLa cells were transfected with poly(dA:dT) for the indicated times and treated with MG132 for 6 hr before cell harvest. Data are representative of at least three independent experiments. Data are representative of at least three independent experiments. (G) Immunoblot analysis of ubiquitinated IFI16 in control HeLa cells or HPV 18E7 knockout stable HeLa cells transfected with poly(dA:dT) for the indicated times and treated with MG132 for 6 hr before cell harvest. Data are representative of at least three independent experiments.

The data described above showed that the interaction between HPV E7 and IFI16 was reduced in response to poly(dA:dT) transfection. Therefore, we hypothesized that the degradation of IFI16 by HPV E7 might cause the decreased interaction between HPV E7 and IFI16 after poly(dA:dT) transfection. To verify this hypothesis, we detected the expression of IFI16 by western blotting and found that overexpression of HPV 11E7 or HPV 18E7 promoted the degradation of IFI16 (Figure 3A) and knockdown of HPV 18E7 inhibited the degradation of IFI16 after poly(dA:dT) transfection (Figure 3B). The cycloheximide chase assay showed that overexpression of HPV 18E7 shortened the half-life of IFI16 (Figure 3C) and silencing of HPV 18E7 extended the half-life of the IFI16 protein in HeLa cells in response to poly(dA:dT) transfection (Figure 3D). To further confirm the role of HPV E7 in regulating the protein level of IFI16, we cotransfected 293T cells with plasmids for Flag-IFI16 and GFP-HPV 18E7 expression and observed that HPV 18E7 could also promote the degradation of IFI16 (Figure 3E). However, the degradation of IFI16 induced by HPV 18E7 was blocked by MG132, which indicated that the degradation of IFI16 induced by HPV E7 was dependent on the ubiquitin-proteasome system (Figure 3E). Next, we explored whether HPV 18E7 affected the ubiquitination of IFI16. HPV 18E7-silenced HeLa cells displayed decreased ubiquitination of IFI16 after poly(dA:dT) transfection (Figure 3F). We also found that overexpression of HPV 18E7 promoted the ubiquitination of IFI16 (Figure 3G). These data demonstrated that HPV E7 promotes the ubiquitin-mediated degradation of IFI16.

### IFI16 is responsible for the pyroptosis induced by intracellular dsDNA

**Figure 4:**
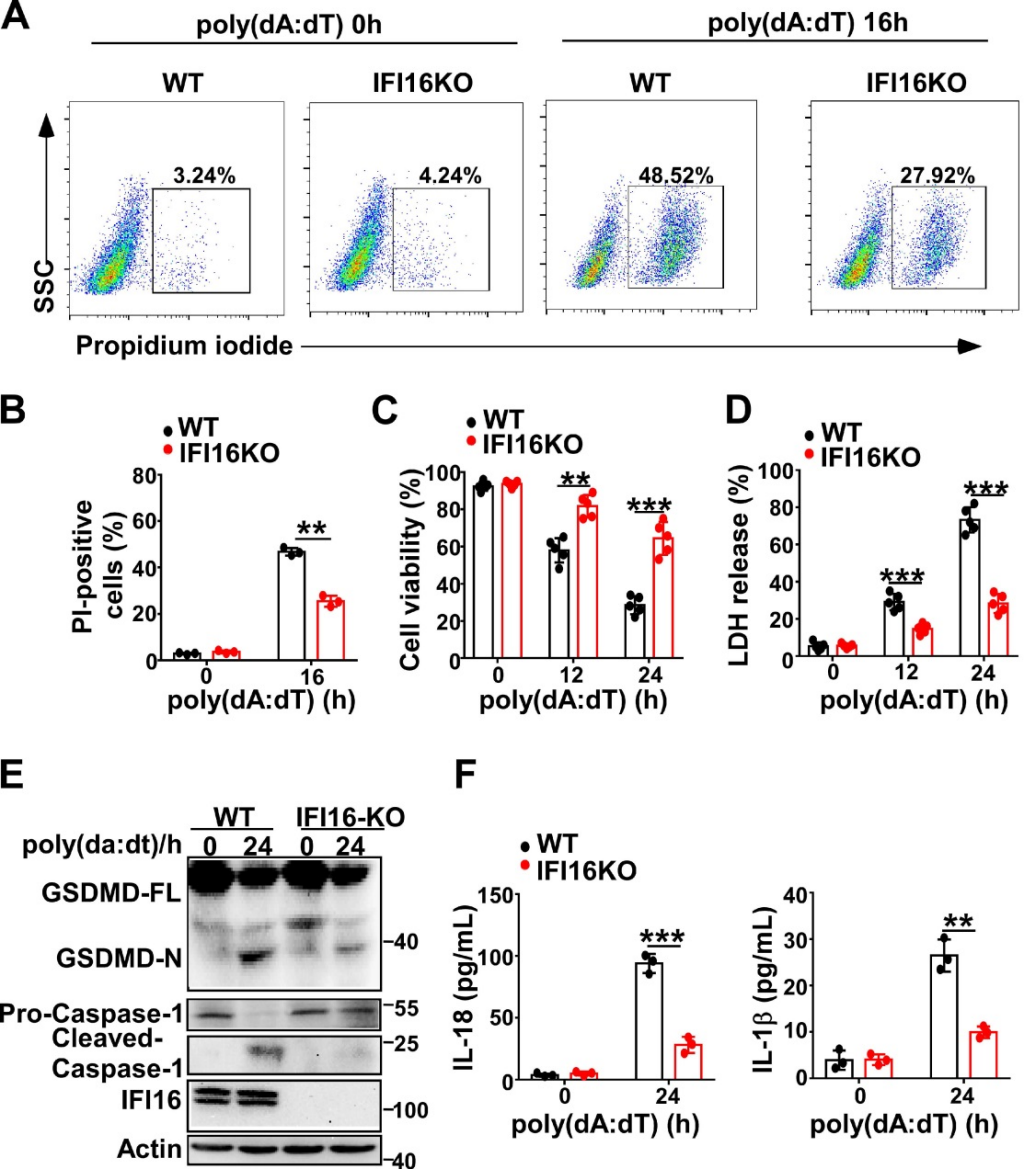
** IFI16 is responsible for pyroptosis induced by intracellular dsDNA.** (A) Microscopy imaging showing cell death in IFI16 knockout stable HeLa cells or control HeLa cells transfected with poly(dA:dT) for 18 hr. (B) Flow cytometry analysis of propidium iodide-positive control HeLa cells or IFI16 knockout stable HeLa cells transfected with poly(dA:dT) for 16 hr. Cell viability assay(C) and LDH assay (D) of control HeLa cells or IFI16 knockout stable HeLa cells transfected with poly(dA:dT) for the indicated times. (E) Immunoblot analysis of GSDMD and caspase-1 in the lysates of control HeLa cells or IFI16 knockout stable HeLa cells transfected with poly(dA:dT) for 24 hr. (F) ELISA analysis of IL-18 and IL-1β in control HeLa cells or IFI16 knockout stable HeLa cell transfected with poly(dA:dT) for 24 hr. Data are presented as mean ± SD of duplicate samples and are representative of at least three independent experiments. *P* values are determined by two-tailed Student's *t* test. **p < 0.01, ***p < 0.001.

Next, we investigated whether IFI16 played a role in pyroptosis induced by intracellular dsDNA. Knockout of IFI16 by CRISPR/Cas9 significantly decreased the propidium iodide-positive HeLa cells after transfection with poly(dA:dT) (Figure 4A-B). IFI16KO HeLa cells displayed higher cell viability than WT HeLa cells (Figure 4C). However, the LDH level in IFI16KO HeLa cells was lower than that in WT HeLa cells after transfection with poly(dA:dT) (Figure 4D). Furthermore, knockout of IFI16 inhibited the cleavage of GSDMD and the activation of caspase-1 in HeLa cells (Figure 4E). ELISA assays showed that the levels of IL-1β and IL-18 were significantly decreased in IFI16KO HeLa cells transfected with poly(dA:dT) (Figure 4F). Taken together, our results demonstrated that IFI16 is responsible for the pyroptosis induced by intracellular dsDNA.

### HPV E7 interacts with IFI16 and E3 ligaseTRIM21

**Figure 5:**
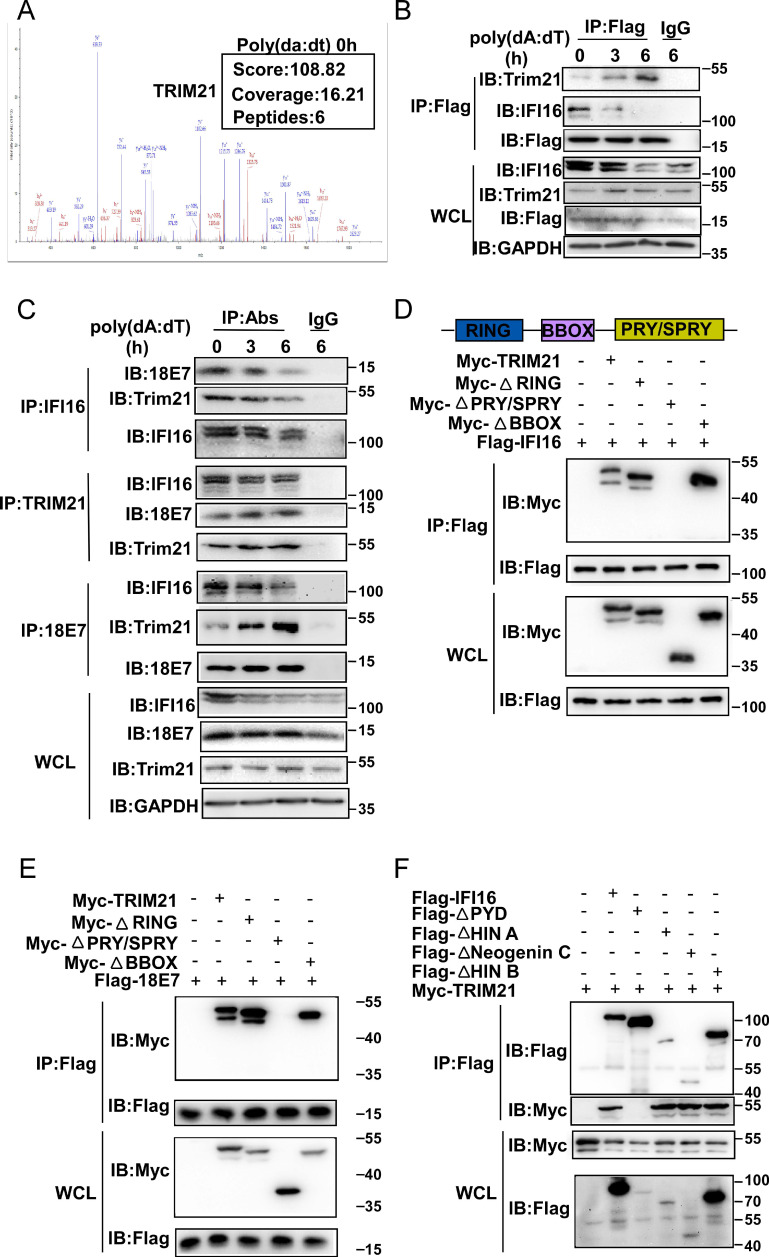
** HPV E7 interacts with IFI16 and the E3 ligaseTRIM21.** (A) Mass spectrum data of TRIM21 among HPV 11E7-interacting proteins identified by mass spectrometry. (B) Immunoblot analysis of HaCaT cells stably expressing HPV 11E7 transfected with poly(dA:dT) for the indicated times, followed by immunoprecipitation with anti-FlagM2 beads. (C) Immunoblot analysis of HeLa cells transfected with poly(dA:dT) for the indicated times, followed by immunoprecipitation with IFI16, HPV 18E7, TRIM21 or immunoglobulin G (IgG)-conjugated magnetic beads. (D) Coimmunoprecipitation and immunoblot analysis of 293T cells cotransfected for 36 hr with Flag-IFI16 plus Myc-TRIM21 or Myc-TRIM21 mutant vectors, followed by immunoprecipitation with anti-Flag-M2 beads. (E) Coimmunoprecipitation and immunoblot analysis of 293T cells cotransfected for 36 hr with Flag-HPV 18E7 plus Myc-TRIM21 or Myc-TRIM21 mutant vectors, followed by immunoprecipitation with anti-FlagM2 beads. (F) Coimmunoprecipitation and immunoblot analysis of 293T cells cotransfected for 36 hr with Myc-TRIM21 and Flag-IFI16 or Flag-IFI16 mutant vectors, followed by immunoprecipitation with anti-FlagM2 beads.

Our results demonstrated that HPV E7 promoted the ubiquitin-proteasome-mediated degradation of IFI16. The ubiquitin-proteasome system is mainly composed of ubiquitin-activating enzyme (E1), ubiquitin-crosslinking enzyme (E2), ubiquitin ligase (E3) and the 26S proteasome 32. Among these components, the E3 ubiquitin ligase is responsible for target protein recognition and mediates the degradation of substrates by the 26S proteasome 33. To determine which E3 ubiquitin ligase mediated the degradation and ubiquitination of IFI16, we analyzed data from the above mass spectrometry experiment. Among the HPV 11E7-interacting proteins, the outstanding mascot scores indicated that TRIM21 could interact with HPV 11E7 (Figure 5A). To confirm the interaction between TRIM21, HPV E7, and IFI16, we immunoprecipitated Flag-HPV 11E7 from the lysates of cells overexpressing Flag-HPV 11E7; immunoblotting revealed that the interaction between HPV 11E7 and TRIM21 was increased in HaCaT cells after poly(dA:dT) transfection (Figure 5B). Additionally, the interaction between HPV 18E7 and TRIM21 was increased in HeLa cells during poly(dA:dT) transfection (Figure 5C). Further coimmunoprecipitation and immunoblot assays showed that HPV 18E7 and IFI16 interacted with TRIM21 in 293T cells transfected with plasmids expressing TRIM21, IFI16 or HPV 18E7 (Figure S3A-S3B). Additionally, HPV E7 promoted the interaction between IFI16 and TRIM21 (Figure S3C).

TRIM21 contains RING, BBOX, and PRY/SPRY domains 34. To identify which domain of TRIM21 was responsible for the interaction with HPV E7 or IFI16, we constructed mutant TRIM21 plasmids and found that depletion of the PRY/SPRY domain eliminated the interactions of TRIM21 with HPV 18E7 and IFI16 (Figure 5D-5E). Furthermore, we found that TRIM21 interacted with the PYD domain of IFI16 (Figure 5F). These results strongly suggested that TRIM21 can interact with IFI16 and HPV E7.

### HPV E7 promotes the K33-linked ubiquitination and degradation of IFI16 mediated by TRIM21

**Figure 6:**
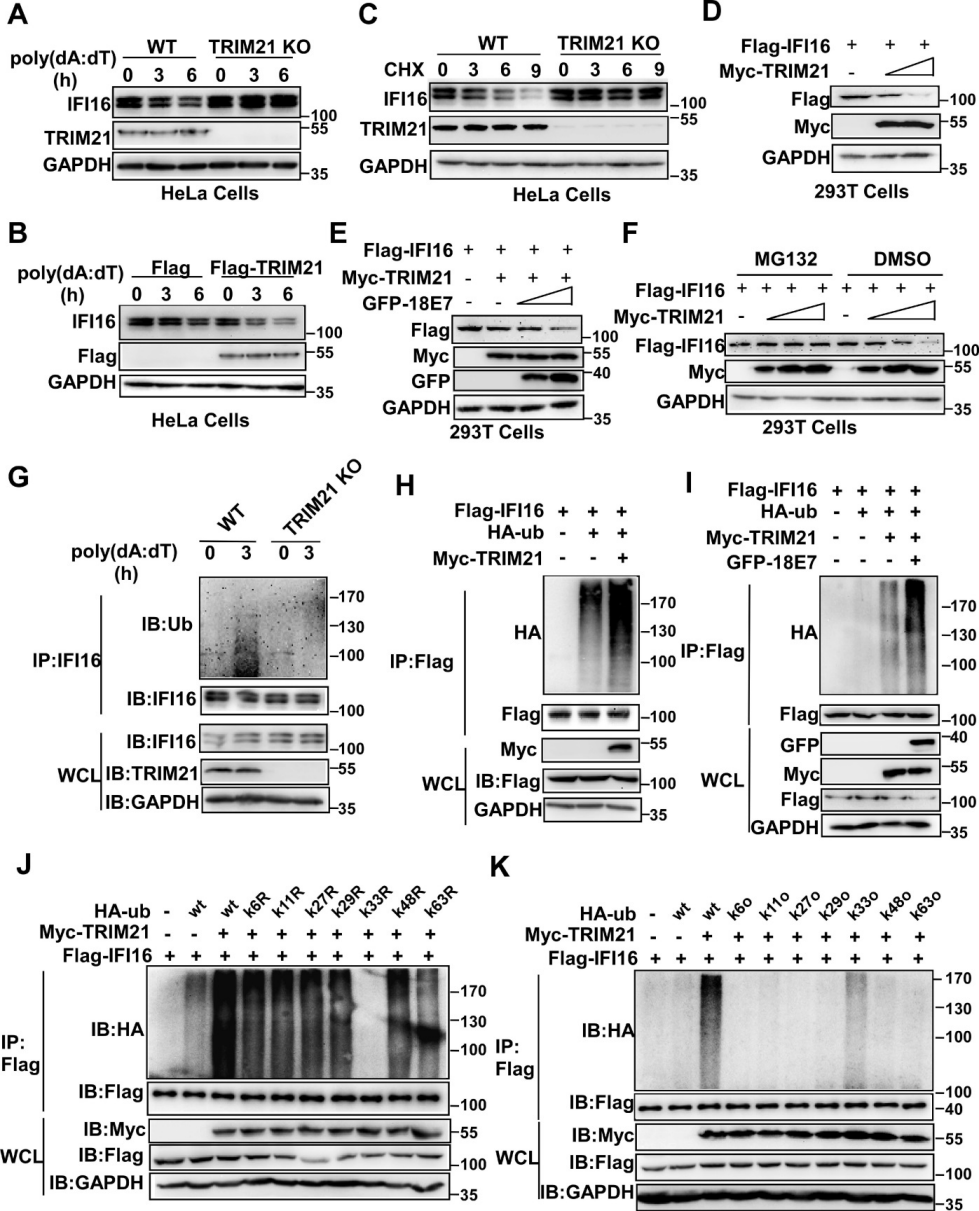
** HPV E7 promotes the K33-linked ubiquitination and degradation of IFI16 mediated by TRIM21.** (A) Immunoblot analysis of lysates in control HeLa cells or TRIM21 knockout stable HeLa cells transfected with poly(dA:dT) for the indicated times. (B) Immunoblot analysis of IFI16 in the lysates of TRIM21 knockout stable HeLa cells treated with CHX (40 μg/ml) for the indicated number of hours after transfection with poly(dA:dT) for 1 hr. (C) Immunoblot analysis of 293T cells cotransfected with the Myc-TRIM21 and Flag-IFI16 vectors for 36 hr. (D) Immunoblot analysis of 293T cells cotransfected with the Myc-TRIM21 and Flag-IFI16 vectors with or without MG132 treatment. (E) Immunoblot analysis of 293T cells cotransfected with the Myc-TRIM21 and Flag-IFI16 vectors treated with CHX (40 μg/ml) for the indicated number of hours. (F) Immunoblot analysis of 293T cells cotransfected with Myc-TRIM21and the Flag-IFI16 or GFP-HPV 18E7 vector for 36 hr. (G) Immunoblot analysis of ubiquitinated IFI16 in control HeLa cells or TRIM21 knockout stable HeLa cells transfected with poly(dA:dT) for the indicated times and treated with MG132 for 6 hr before cell harvest. (H) Immunoblot analysis of ubiquitinated IFI16 in 293T cells cotransfected with Myc-TRIM21and the Flag-IFI16 or HA-ub vector for 36 hr with or without MG132 treatment for 6 hr before cell harvest. (I) Immunoblot analysis of ubiquitinated IFI16 in 293T cells transfected with combinations of the Myc-TRIM21, Flag-IFI16, HA-ub, and GFP-HPV 18E7 vectors for 36 hr and treated with MG132 for 6 hr before cell harvest. (J) Immunoblot analysis of ubiquitinated IFI16 in 293T cells transfected with combinations of the Myc-TRIM21, Flag-IFI16, HA-ub, and HA-ub mutant (each of the Lys residues were replaced by an Arg residue) vectors for 36 hr and treated with MG132 for 6 hr before cell harvest. (K) Immunoblot analysis of ubiquitinated IFI16 in 293T cells transfected with combinations of the Myc-TRIM21, Flag-IFI16, HA-ub, HA-ub mutant (all Lys residues but one was replaced with Arg residues) vectors for 36 hr and treated with MG132 for 6 hr before cell harvest. Data are representative of at least three independent experiments.

Next, we investigated whether TRIM21 mediated the ubiquitination and degradation of IFI16. The level of IFI16 was higher in TRIM21KO HeLa cells than in WT HeLa cells in response to poly(dA:dT) transfection (Figure 6A). Overexpression of TRIM21 promoted the degradation of IFI16 in HeLa cells after poly(dA:dT) transfection (Figure 6B). The cycloheximide chase assay showed that deletion of TRIM21 extended the half-life of the IFI16 protein (Figure 6C). Overexpression of TRIM21 shortened the half-life of IFI16 in HeLa cells (Figure S4A) or 293T cells (Figure S4B) transfected with poly(dA:dT). Furthermore, TRIM21 inhibited IFI16 protein levels when we cotransfected 293T cells with plasmids expressing Flag-IFI16 and Myc-TRIM21 (Figure 6D). Moreover, HPV 18E7 promoted the degradation of IFI16 mediated by TRIM21 (Figure 6E). However, this reduction in IFI16 mediated by TRIM21 was blocked by MG132, which implies that TRIM21 mediates the degradation of IFI16 by the ubiquitin-proteasome system (Figure 6F). Our data indicated that HPV E7 promotes the degradation of IFI16 mediated by TRIM21.

Next, we explored whether TRIM21 mediated the ubiquitination of IFI16. Knockout of TRIM21 deceased the ubiquitination of IFI16 in HeLa cells after poly(dA:dT) transfection (Figure 6G). The ubiquitination of IFI16 was increased in TRM21-overexpressing HeLa cells after poly(dA:dT) transfection (Figure S4C). The TRIM21-mediated ubiquitination of IFI16 was also detected in 293T cells transfected with Flag-IFI16, HA-ub, and Myc-TRIM21 (Figure 6H). Furthermore, HPV 18E7 promoted the ubiquitination of IFI16 mediated by TRIM21 (Figure 6I). Ubiquitin contains seven lysine (K) residues (K6, K11, K27, K29, K33, K48, and K63) that are utilized to form ubiquitination chains 35. To determine which form of polyubiquitin chain was attached to IFI16 by TRIM21, we mutated each of the Lys residues on ubiquitin and tested their effects on IFI16 ubiquitination in 293T cells overexpressing TRIM21 and IFI16. Only K33R ubiquitin did not support TRIM21-mediated IFI16 ubiquitination (Figure 6J). We next used another set of ubiquitin mutants in which all but one Lys residue were replaced with an Arg. Among these ubiquitin mutants, only K33-ubiquitin and wild-type ubiquitin promoted ubiquitin chain formation on IFI16 (Figure 6K). Taken together, our results demonstrated that HPV E7 can promote the K33-linked ubiquitination and degradation of IFI16 mediated by TRIM21.

### TRIM21 downregulates cell pyroptosis induced by poly(dA:dT)

**Figure 7:**
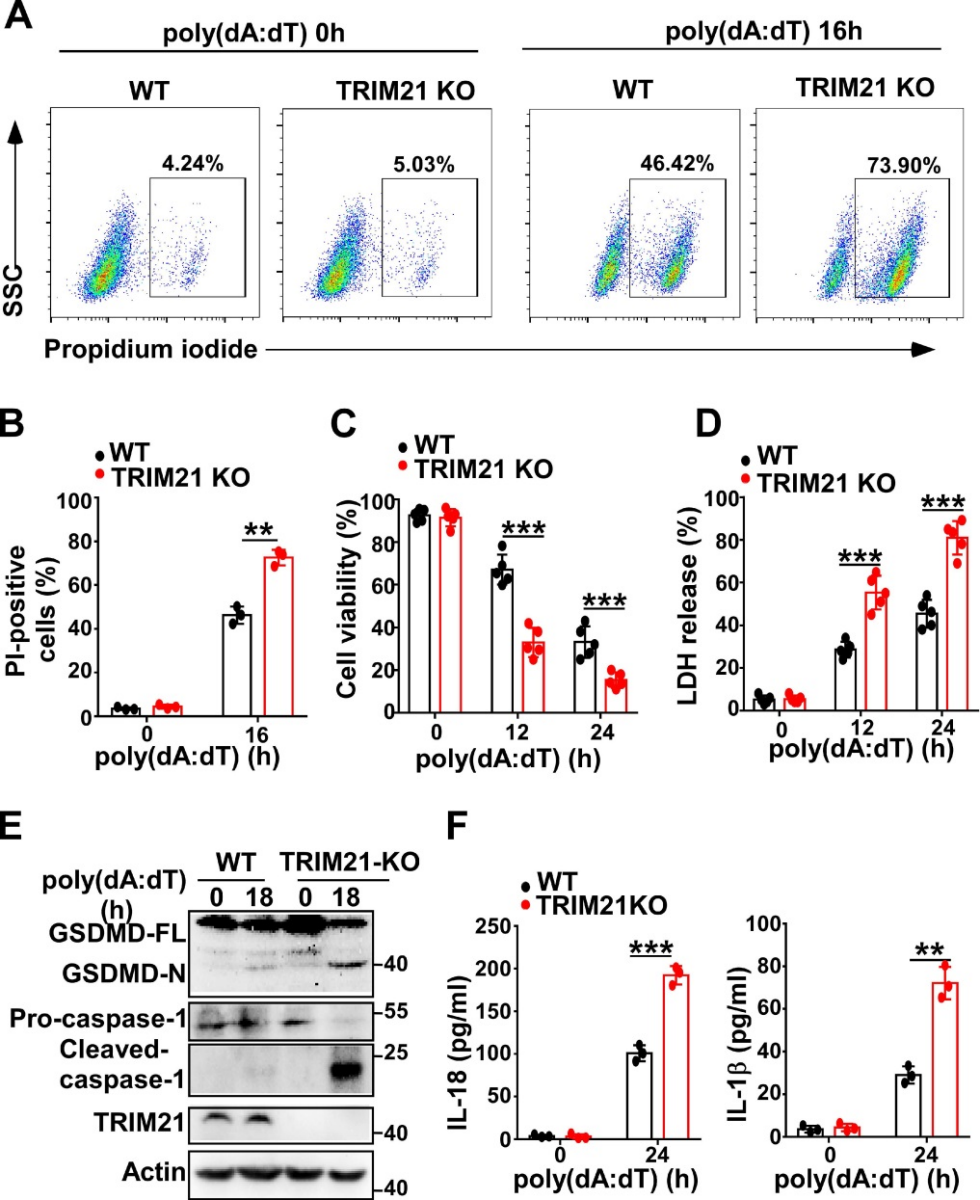
** TRIM21 downregulates cell pyroptosis induced by poly(dA:dT).** (A) Microscopy imaging of cell death in control HeLa cells or TRIM21 knockout stable HeLa cells transfected with poly(dA:dT) for 18 hr. (B) Flow cytometry analysis of propidium iodide-positive control HeLa cells or TRIM21 knockout stable HeLa cells transfected with poly(dA:dT) for 16 hr. (C) Cell viability assay in control HeLa cells or TRIM21 knockout stable HeLa cells transfected with poly(dA:dT) for the indicated times. (D) LDH assay in control HeLa cells or TRIM21 knockout stable HeLa cells transfected with poly(dA:dT) for the indicated times. (E) Immunoblot analysis of GSDMD and caspase-1 in the lysates of control HeLa cells or TRIM21 knockout stable HeLa cells transfected with poly(dA:dT) for 24 hr. (F) ELISA analysis of IL-18 and IL-1β in control HeLa cells or knockout stable HeLa cells transfected with poly(dA:dT) for 24 hr. Data are presented as mean ± SD of duplicate samples and are representative of at least three independent experiments. P values are determined by two-tailed Student's *t* test. **p < 0.01, ***p < 0.001.

Next, we investigated whether TRIM21 could regulate pyroptosis induced by poly(dA:dT). Flow cytometry also showed that more TRIM21 KO HeLa cells than control cells were propidium iodide-positive in response to transfection with poly(dA:dT) (Figure 7A-B). In addition, TRIM21KO HeLa cells displayed decreased cell viability compared to WT HeLa cells in response to poly(dA:dT) (Figure 7C). Furthermore, knockout of TRIM21 significantly promoted the release of LDH in HeLa cells (Figure 7D). Moreover, immunoblotting showed that the level of GSDMD-N and activated caspase-1 were higher in TRIM21KO HeLa cells than in WT HeLa cells after transfection with poly(dA:dT) (Figure 7E). Knockout of TRIM21 significantly enhanced the production of IL-1β and IL-18 in HeLa cells transfected with poly(dA:dT) (Figure 7F). Thus, our results indicated that TRIM21 inhibits pyroptosis induced by poly(dA:dT).

## Discussion

Host cell death, including apoptotic cell death, necrotic cell death, and pyroptotic cell death, is a critical immune defense mechanism in response to infection with pathogenic organisms 36. However, HPV has evolved mechanisms to manipulate host cell death to enhance its ability to survive, evade immunosurveillance and cause disease 37, 38. For example, the HPV E5 protein can protect cells from tumor necrosis factor-related apoptosis 39, HPV E6 can inhibit apoptosis mainly through the p53 pathway 40, and HPV E7 plays a role in both apoptosis activation and inhibition 41. However, whether HPV can regulate cell pyroptosis is unknown. Here, we found that HPV 11E7 or HPV 18E7 could inhibit the activation of caspase-1 and cleavage of GSDM-D. HPV E7 could also attenuate cell swelling and the production of IL-18 and IL-1β which can promote the production of proinflammatory cytokines from immune cells and enhance the expansion, migration, and activation of macrophages, neutrophils, CD8^+^ T cells, Th17 cells and NK cells during infections 42. Therefore, HPV could evade immunosurveillance and cause cervical cancer or condyloma acuminate by regulating cell pyroptosis. Our data reveals one of the mechanisms of HPV immune escape.

IFI16 is required for the innate immune response to transfected DNA and infection with nuclear and cytosolic DNA viruses 24, 43. IFI16 and cGAS cooperate to sense intracellular DNA and enable the optimal production of cGAMP, which activates the STING-TBK1 pathway and induces the production of type I interferon in human keratinocytes or macrophages 23, 24. IFI16 can detect viral DNA, initiate assembly of the inflammasome and mediate cell pyroptosis upon infection with DNA viruses, such as KSHV, EBV or HSV-1 44. IFI16 inhibits HPV18 replication by repressing viral gene expression and replication 45. Numerous studies have shown that pathogens promote immune escape by regulating the expression and function of IFI16 through mechanisms such as production of the HSV-1-encoded infected cell protein (ICP)0 protein, which possesses E3 ubiquitin ligase activity and mediates the degradation of IFI16 by the proteasome 28. KSHV selectively degrades IFI16 to evade the host immune system to maintain latency 46. The HCMV major tegument protein pUL83 interacts with IFI16 and inhibits the oligomerization of activated IFI16, resulting in diminished signal transmission from IFI16 to the STING-TBK1-IRF3 signaling pathway 47. Our results demonstrated that HPV E7 interacted with the HINB domain of IFI16 and promoted the ubiquitin-proteasome-mediated degradation of IFI16 by recruiting the E3 ligase TRIM21, resulting in the inhibition of cell pyroptosis during infection.

Ubiquitination is a dynamic, multifaceted posttranslational modification that plays a pivotal role in the regulation of many cellular processes including proteasomal degradation, the cell cycle, DNA metabolism, and various signal transduction pathways 48. Many viruses including HPV usurp ubiquitination and ubiquitin-like modifications to reprogram the cellular environment to favor viral persistence and reproduction 49. High-risk HPV E6 protein is the prototype of HPV-mediated modulation of the host cell through recruiting the ubiquitin ligase E6AP to mediate the ubiquitination and degradation of p53 50. HPV16 E7 promotes the ubiquitination and degradation of pRB mediated by the ubiquitin ligase cullin 2 and the E6-pRb complex 51. High-risk HPV E7 limits keratinocyte differentiation and contributes to HPV-mediated oncogenesis by facilitating E3 ligase UBR4-mediated ubiquitination and the degradation of PTPN14 52. The HPV E6 and E7 suppress IRF3 activation by inhibiting K63-linked ubiquitination of TRAF3 through upregulating UCHL1 and inhibiting the immune response upon HPV infection 53. Here, we found that the interaction between HPV E7 and TRIM21 was increased after transfection with poly(dA:dT). It has been reported that the phosphorylation of TRIM21 can be catalyzed by IKKβ or TBK1 kinases, thus releasing the auto-inhibitory mechanism of TRIM21 54. Therefore, the increased interaction of HPV E7 and TRIM21 may be due to the increased phosphorylation of TRIM21 catalyzed by IKKβ or TBK1 kinases upon poly(dA:dT) transfection. We also found that the interaction between HPV E7, TRIM21 and IFI16 was significantly decreased after transfection with poly(dA:dT). This implied that HPV E7 could recruit the E3 ligase TRIM21 to mediate the ubiquitination and degradation of IFI16 during HPV infection.

**Figure 8:**
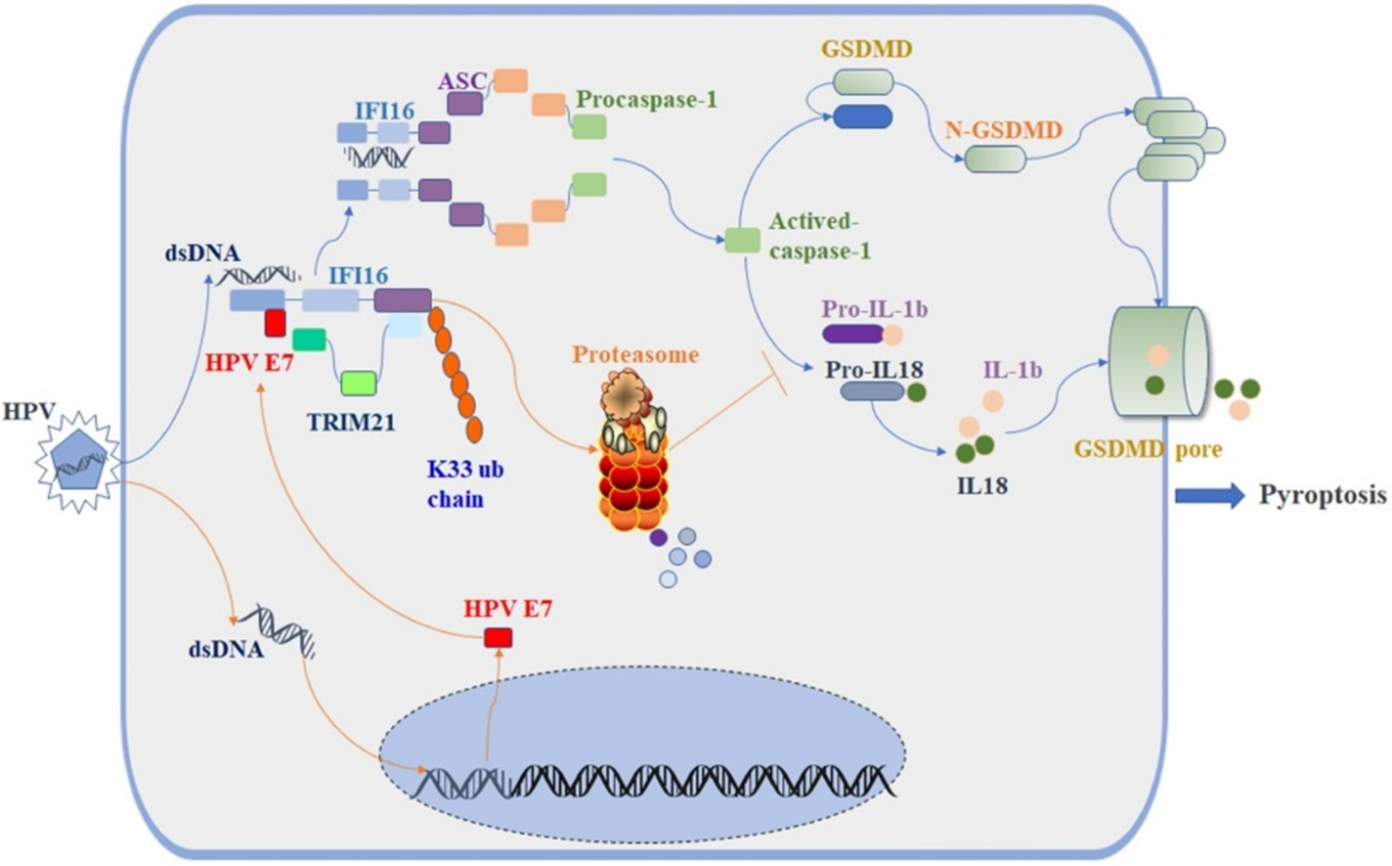
Schematic diagram showing that HPV E7 interacted with IFI16 and promoted the ubiquitin-mediated degradation of IFI16 by recruiting the E3 ligase TRIM21, resulting in the inhibition of cell pyroptosis during HPV infection.

The E3 ligase TRIM21 is a member of the TRIM protein family largely involved in innate immunity 55. TRIM21 mediates the polyubiquitination and degradation of the DNA sensor DDX41 and the IRF transcription factors IRF3, IRF5, and IRF7 to negatively regulate the production of interferon-β during viral infection 56. Recent research reported that TRIM21 can also promote the STING-mediated IFI16 degradation and negatively regulate the production of type I interferon 57. However, a growing number of studies have shown that TRIM21 positively upregulates type I IFN signaling through promoting cGAS and RIG-I sensing of viral genomes or interfering with the degradation of IRF3 and IRF8 58, 59. Therefore, the role of TRIM21 in antiviral innate immunity remains controversial, and it is unclear whether TRIM21 plays a role in inflammasome-mediated cell pyroptosis. In this study, we found the proportion of swollen cells, the level of GSDMD-N and the activated caspase-1 were higher in TRIM21KO HeLa cells than in WT HeLa cells after transfection with poly(dA:dT). Knockout of TRIM21 significantly inhibited the production of IL-1β and IL-18. Moreover, the regulatory effect of HPV 18E7 on pyroptosis and the stability of IFI16 were absent in TRIM21 KO HeLa cells transfected with poly(dA:dT). Our results indicated that TRIM21 negatively regulated the activation of the IFI16 inflammasome and that HPV E7-mediated regulation of pyroptosis was dependent on TRIM21. Therefore, based on the results of our study, we have schematically drawn a diagraph to show the effect and mechanisms of HPV E7 on cell pyroptosis (Figure 8).

## Supplementary Material

Supplementary figures and tables.
