# Supplementary material for: HPV E7 inhibits cell pyroptosis by promoting TRIM21-mediated degradation and ubiquitination of the IFI16 inflammasome
Source: Int J Biol Sci. 2020 Sep 13;16(15):2924–37. doi: 10.7150/ijbs.50074 (PMC7545706; doi:10.7150/ijbs.50074)
Supplement: Supplementary file 1 — Supplementary figures and tables. [file ijbsv16p2924s1.pdf]

# Supplementary information:

## Supplementary figure 1

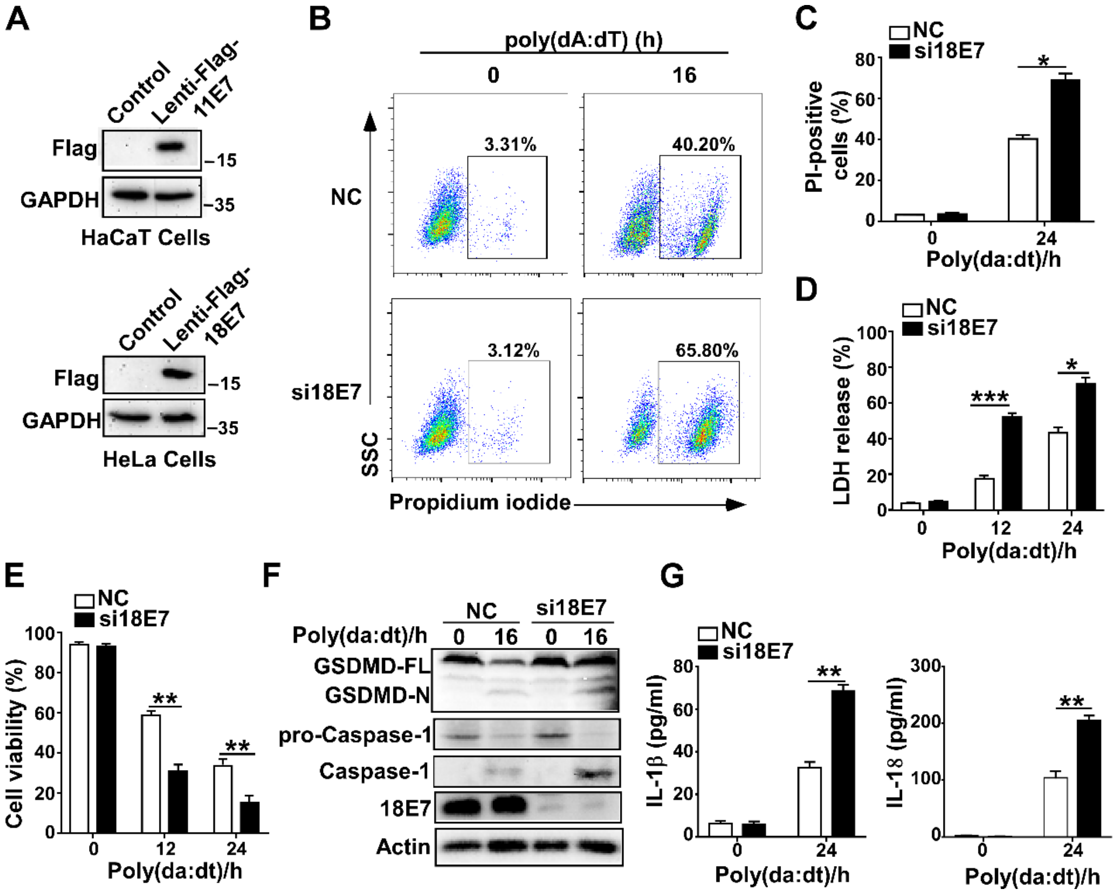


**Figure S1. HPV E7 inhibits pyroptosis induced by intracellular dsDNA.** (A) Immunoblot analysis of HPV E7 in HaCaT cells stably expressing HPV11E7 or HeLa cells stably expressing HPV 18E7. (B-C) Flow cytometry analysis (B) and statistical analysis (C) of propidium iodide-positive control or HPV 18E7 silenced HeLa cells after transfection with poly(dA:dT) for the indicate times. (D-E) LDH assay (D) and Cell viability assay (E) to detect dead control or HPV 18E7 silenced HeLa cells after transfection with poly(dA:dT) for the indicate times. (F) Immunoblot analysis of GSDMD and caspase-1 in the lysates of control or HPV 18E7 silenced HeLa cells transfected with poly(dA:dT) for 16 hr. (G) ELISA analysis of IL-18 and IL-1β in control or HPV 18E7 silenced HeLa cells transfected with poly(dA:dT) for 24 hr. Data are presented as mean ± SD of duplicate samples and are representative of at least three independent experiments. P values are determined by two-tailed Student's *t* test. *$p < 0.05$, **$p < 0.01$, ***$p < 0.001$.

**Supplementary figure 2**

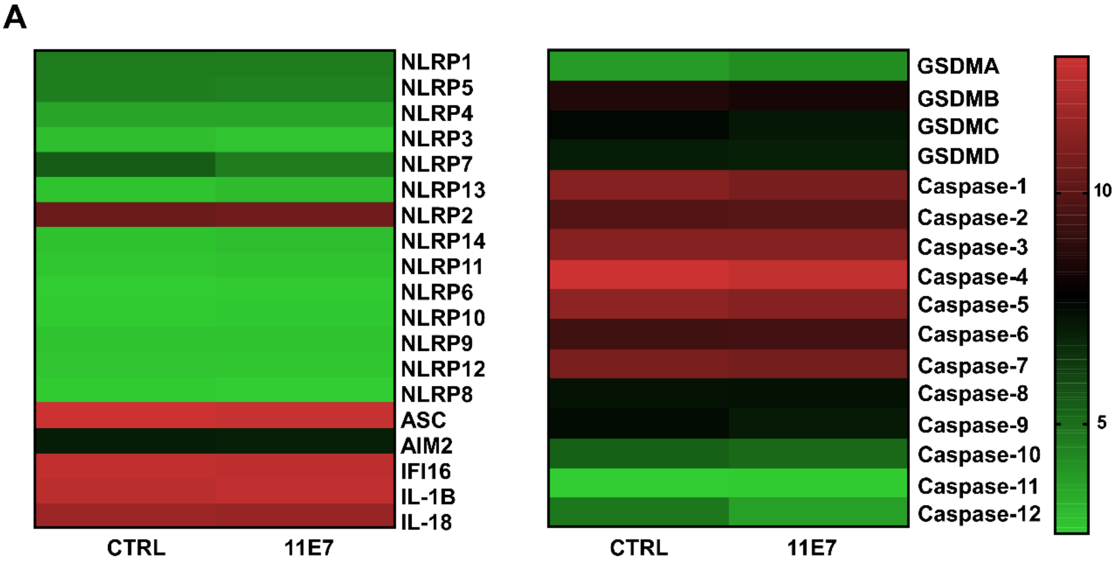


**Figure S2. HPV E7 does not affect the mRNA level of genes associated with pyroptosis.** (A) RNA-seq analysis of HaCaT cells stably expressing HPV 11E7. Data are representative of at two independent experiments.

**Supplementary figure 3**

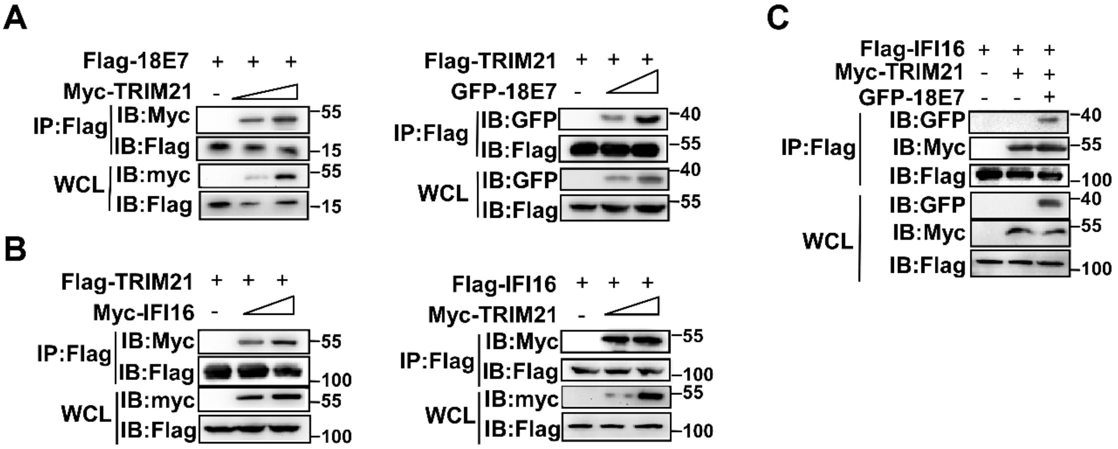


**Figure S3. HPV E7 interacts with IFI16 and the E3 ligaseTRIM21.** (A) Coimmunoprecipitation and immunoblot analysis of 293T cells cotransfected for 36 h with Flag-18E7 and Myc-TRIM21 or Flag-TRIM21 and GFP-18E7, followed by immunoprecipitation with anti-FlagM2 beads. (B) Coimmunoprecipitation and immunoblot analysis of 293T cells cotransfected for 36h with Flag-TRIM21 and Myc-IFI16 or Flag-IFI16 and Myc-TRIM21, followed by immunoprecipitation with anti-FlagM2 beads. (C) Coimmunoprecipitation and immunoblot analysis of 293T cells cotransfected for 36 h with

Flag-IFI16 and Myc-TRIM21 or GFP-18E7, followed by immunoprecipitation with anti-FlagM2 beads. Data are representative of at least three independent experiments.

**Supplementary figure 4**

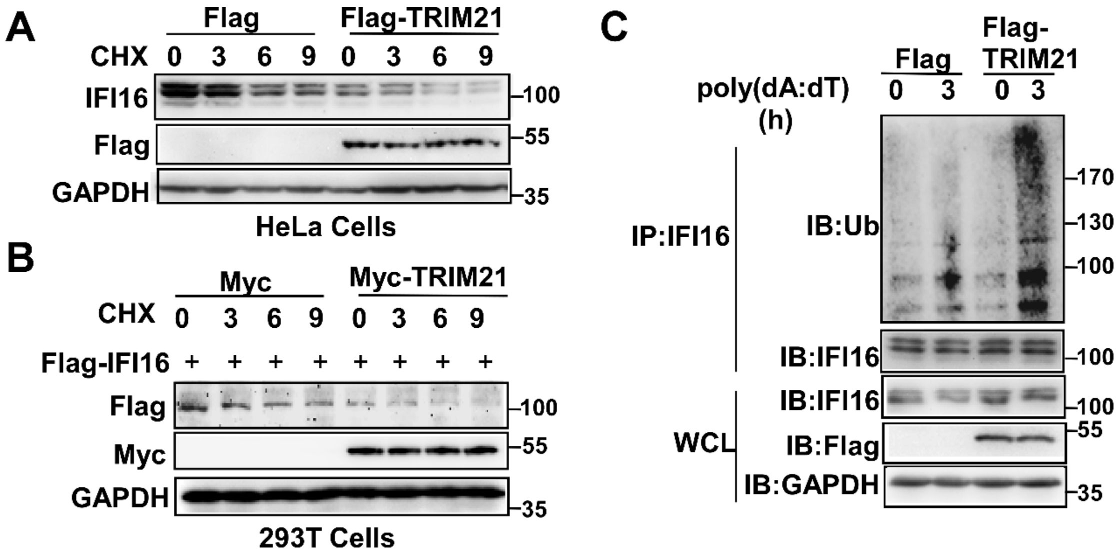


**Figure S4. HPV E7 interacts with IFI16 and the E3 ligaseTRIM21.** (A) Immunoblot analysis of control HeLa cells or TRIM21-overexpressing HeLa cells transfected with poly(dA:dT) for the indicated times. (B) Immunoblot analysis of control HeLa cells or TRIM21-overexpressing HeLa cells treated with CHX (40 μg/ml) for the indicated times after transfection with poly(dA:dT) for 1 hr. (C) Immunoblot analysis of ubiquitinated IFI16 in control HeLa cells or TRIM21-overexpressing HeLa cells transfected with poly(dA:dT) for the indicated times and treated with MG132 for 6 hours before cell harvest. Data are representative of at least three independent experiments.

**Supplementary table 1. The information of HPV 11E7 interacted proteins in stable expression of HPV 11E7 HaCaT cells.**

| Accession | Description | Score | Coverage |
|---|---|---|---|
| B4DTN0 | cDNA FLJ51085, highly similar to Retinoblastoma-associated protein OS=Homo sapiens PE=2 SV=1 - [B4DTN0_HUMAN] | 111.86 | 15.10 |
| P28749 | Retinoblastoma-like protein 1 OS=Homo sapiens GN=RBL1 PE=1 SV=3 - [RBL1_HUMAN] | 95.77 | 17.42 |
| Q9Y2W1 | Thyroid hormone receptor-associated protein 3 OS=Homo sapiens GN=THRAP3 PE=1 SV=2 - [TR150_HUMAN] | 92.02 | 11.73 |

| | | | |
|---|---|---|---|
| P19474 | E3 ubiquitin-protein ligase TRIM21 OS=Homo sapiens GN=TRIM21 PE=1 SV=1 - [RO52_HUMAN] | 91.99 | 10.11 |
| Q86V81 | THO complex subunit 4 OS=Homo sapiens GN=ALYREF PE=1 SV=3 - [THOC4_HUMAN] | 71.23 | 17.12 |
| Q6P2Q9 | Pre-mRNA-processing-splicing factor 8 OS=Homo sapiens GN=PRPF8 PE=1 SV=2 - [PRP8_HUMAN] | 67.15 | 5.14 |
| X5D2J9 | General transcription factor IIi isoform D (Fragment) OS=Homo sapiens GN=GTF2I PE=2 SV=1 - [X5D2J9_HUMAN] | 58.09 | 13.27 |
| Q7Z406 | Myosin-14 OS=Homo sapiens GN=MYH14 PE=1 SV=2 - [MYH14_HUMAN] | 55.18 | 4.41 |
| Q96DI9 | POLDIP3 protein (Fragment) OS=Homo sapiens GN=POLDIP3 PE=2 SV=2 - [Q96DI9_HUMAN] | 53.69 | 19.66 |
| A0A024RAR0 | Proline-rich protein HaeIII subfamily 1, isoform CRA_a OS=Homo sapiens GN=PRH1 PE=4 SV=1 - [A0A024RAR0_HUMAN] | 49.31 | 45.78 |
| Q8TB01 | Similar to cytoskeleton-associated protein 4 (Fragment) OS=Homo sapiens PE=2 SV=1 - [Q8TB01_HUMAN] | 41.41 | 15.71 |
| Q96RE1 | Translation elongation factor 1 alpha 1-like 14 OS=Homo sapiens GN=EEF1A1L14 PE=2 SV=1 - [Q96RE1_HUMAN] | 41.32 | 14.82 |
| K7EK45 | Polypyrimidine tract-binding protein 1 (Fragment) OS=Homo sapiens GN=PTBP1 PE=1 SV=1 - [K7EK45_HUMAN] | 40.41 | 23.05 |
| B7Z3V1 | Sodium/potassium-transporting ATPase subunit alpha (Fragment) OS=Homo sapiens PE=2 SV=1 - [B7Z3V1_HUMAN] | 40.36 | 8.04 |
| B4DSZ9 | E3 ubiquitin-protein ligase TTC3 OS=Homo sapiens GN=TTC3 PE=1 SV=1 - [B4DSZ9_HUMAN] | 37.11 | 2.61 |
| A0A024R1X8 | Junction plakoglobin, isoform CRA_a OS=Homo sapiens GN=JUP PE=4 SV=1 - [A0A024R1X8_HUMAN] | 36.17 | 8.59 |
| P30050 | 60S ribosomal protein L12 OS=Homo sapiens GN=RPL12 PE=1 SV=1 - [RL12_HUMAN] | 36.00 | 28.48 |
| F6IB50 | MHC class I antigen (Fragment) OS=Homo sapiens GN=HLA-A PE=4 SV=1 - [F6IB50_HUMAN] | 35.30 | 14.13 |
| E5RI99 | 60S ribosomal protein L30 (Fragment) OS=Homo sapiens GN=RPL30 PE=1 SV=1 - [E5RI99_HUMAN] | 32.33 | 24.56 |
| B2RAM6 | Kinesin-like protein OS=Homo sapiens PE=2 SV=1 - [B2RAM6_HUMAN] | 29.65 | 6.25 |
| Q2VPJ6 | HSP90AA1 protein (Fragment) OS=Homo sapiens | 29.17 | 9.91 |

| | | | |
|---|---|---|---|
| | GN=HSP90AA1 PE=1 SV=1 - [Q2VPJ6_HUMAN] | | |
| Q15208 | Serine/threonine-protein kinase 38 OS=Homo sapiens GN=STK38 PE=1 SV=1 - [STK38_HUMAN] | 28.74 | 17.42 |
| Q8IYY2 | KCTD2 protein (Fragment) OS=Homo sapiens GN=KCTD2 PE=2 SV=1 - [Q8IYY2_HUMAN] | 28.05 | 11.43 |
| A0A0J9YXZ5 | Ras GTPase-activating-like protein IQGAP1 OS=Homo sapiens GN=IQGAP1 PE=1 SV=1 - [A0A0J9YXZ5_HUMAN] | 27.22 | 5.12 |
| Q96BG6 | ACTN4 protein (Fragment) OS=Homo sapiens GN=ACTN4 PE=2 SV=2 - [Q96BG6_HUMAN] | 27.14 | 8.20 |
| E9PPJ5 | Midkine (Fragment) OS=Homo sapiens GN=MDK PE=1 SV=1 - [E9PPJ5_HUMAN] | 25.50 | 19.85 |
| D3DUZ3 | Interferon, gamma-inducible protein 16, isoform CRA_a OS=Homo sapiens GN=IFI16 PE=4 SV=1 - [D3DUZ3_HUMAN] | 25.46 | 7.37 |
| Q5JYR4 | Dolichyl-diphosphooligosaccharide--protein glycosyltransferase subunit 2 (Fragment) OS=Homo sapiens GN=RPN2 PE=1 SV=7 - [Q5JYR4_HUMAN] | 25.38 | 23.95 |
| Q59FC6 | Tumor rejection antigen (Gp96) 1 variant (Fragment) OS=Homo sapiens PE=2 SV=1 - [Q59FC6_HUMAN] | 25.28 | 10.24 |
| Q96HX3 | Dolichyl-diphosphooligosaccharide--protein glycosyltransferase subunit 1 (Fragment) OS=Homo sapiens PE=2 SV=1 - [Q96HX3_HUMAN] | 24.96 | 9.15 |
| F8VSC5 | SCY1-like protein 2 (Fragment) OS=Homo sapiens GN=SCYL2 PE=1 SV=1 - [F8VSC5_HUMAN] | 24.16 | 6.02 |
| Q96PK6 | RNA-binding protein 14 OS=Homo sapiens GN=RBM14 PE=1 SV=2 - [RBM14_HUMAN] | 24.13 | 5.98 |
| Q96BS4 | FBL protein (Fragment) OS=Homo sapiens GN=FBL PE=1 SV=2 - [Q96BS4_HUMAN] | 21.82 | 14.62 |
| F5H837 | Retinoblastoma-like protein 2 (Fragment) OS=Homo sapiens GN=RBL2 PE=1 SV=2 - [F5H837_HUMAN] | 19.58 | 2.50 |
| Q96AF9 | ZYX protein (Fragment) OS=Homo sapiens GN=ZYX PE=2 SV=2 - [Q96AF9_HUMAN] | 19.51 | 8.58 |
| Q9H0A0 | RNA cytidine acetyltransferase OS=Homo sapiens GN=NAT10 PE=1 SV=2 - [NAT10_HUMAN] | 18.59 | 4.78 |
| Q7Z759 | CCT8 protein OS=Homo sapiens GN=CCT8 PE=2 SV=1 - [Q7Z759_HUMAN] | 17.68 | 6.64 |
| A0A087WXU5 | Nucleolar protein of 40 kDa OS=Homo sapiens GN=ZCCHC17 PE=1 SV=1 - [A0A087WXU5_HUMAN] | 17.55 | 18.49 |
| Q9BQA1 | Methylosome protein 50 OS=Homo sapiens | 17.45 | 8.48 |

| | | | |
|---|---|---|---|
| | GN=WDR77 PE=1 SV=1 - [MEP50_HUMAN] | | |
| A8K6H6 | cDNA FLJ76875, highly similar to Homo sapiens protein tyrosine phosphatase, non-receptor type 14 (PTPN14), mRNA OS=Homo sapiens PE=2 SV=1 - [A8K6H6_HUMAN] | 16.70 | 2.36 |
| Q504U8 | EGFR protein OS=Homo sapiens GN=EGFR PE=1 SV=1 - [Q504U8_HUMAN] | 15.87 | 2.66 |
| A0A0S2Z415 | Heat shock 60kDa protein 1 isoform 4 OS=Homo sapiens GN=HSPD1 PE=2 SV=1 - [A0A0S2Z415_HUMAN] | 15.71 | 14.79 |
| F5H2D2 | Prohibitin-2 OS=Homo sapiens GN=PHB2 PE=1 SV=1 - [F5H2D2_HUMAN] | 15.69 | 34.48 |
| X6R700 | Chromatin target of PRMT1 protein OS=Homo sapiens GN=CHTOP PE=1 SV=1 - [X6R700_HUMAN] | 15.65 | 18.83 |
| Q59H46 | Integrin beta (Fragment) OS=Homo sapiens PE=2 SV=1 - [Q59H46_HUMAN] | 15.52 | 3.50 |
| G3V1B3 | 60S ribosomal protein L21 OS=Homo sapiens GN=RPL21 PE=1 SV=1 - [G3V1B3_HUMAN] | 15.30 | 17.24 |
| B3KU66 | cDNA FLJ39263 fis, clone OCBBF2009571, highly similar to ATP-dependent RNA helicase A (EC 3.6.1.-) OS=Homo sapiens PE=2 SV=1 - [B3KU66_HUMAN] | 14.64 | 2.91 |
| Q7Z5X3 | EIF3L protein OS=Homo sapiens GN=EIF3L PE=2 SV=2 - [Q7Z5X3_HUMAN] | 13.60 | 8.51 |
| B3KUD7 | DNA helicase OS=Homo sapiens PE=2 SV=1 - [B3KUD7_HUMAN] | 13.60 | 7.19 |
| Q9BUZ4 | TNF receptor-associated factor 4 OS=Homo sapiens GN=TRAF4 PE=1 SV=1 - [TRAF4_HUMAN] | 13.44 | 16.17 |
| F8WAM2 | T-complex protein 1 subunit eta (Fragment) OS=Homo sapiens GN=CCT7 PE=1 SV=1 - [F8WAM2_HUMAN] | 13.36 | 22.45 |
| Q5JSD2 | Voltage-dependent anion-selective channel protein 2 (Fragment) OS=Homo sapiens GN=VDAC2 PE=1 SV=7 - [Q5JSD2_HUMAN] | 13.34 | 6.67 |
| A0A0D9SFE5 | Lamin B1, isoform CRA_a OS=Homo sapiens GN=LMNB1 PE=1 SV=1 - [A0A0D9SFE5_HUMAN] | 12.81 | 10.94 |
| P62750 | 60S ribosomal protein L23a OS=Homo sapiens GN=RPL23A PE=1 SV=1 - [RL23A_HUMAN] | 12.80 | 21.79 |
| H0YF10 | Histone-binding protein RBBP4 (Fragment) OS=Homo sapiens GN=RBBP4 PE=1 SV=1 - [H0YF10_HUMAN] | 12.51 | 6.28 |
| A8K6A2 | cDNA FLJ77317, highly similar to Homo sapiens | 12.51 | 3.06 |

| | | | |
|---|---|---|---|
| | retinoblastoma binding protein 7 (RBBP7), mRNA OS=Homo sapiens PE=2 SV=1 - [A8K6A2_HUMAN] | | |
| A8KA74 | cDNA FLJ76065 OS=Homo sapiens PE=2 SV=1 - [A8KA74_HUMAN] | 12.24 | 4.90 |
| B4DN87 | cDNA FLJ52569, highly similar to Collagen-binding protein 2 OS=Homo sapiens PE=2 SV=1 - [B4DN87_HUMAN] | 11.32 | 6.80 |
| E5RGH4 | Heterogeneous nuclear ribonucleoprotein H (Fragment) OS=Homo sapiens GN=HNRNPH1 PE=1 SV=1 - [E5RGH4_HUMAN] | 11.22 | 33.00 |
| B3KMA6 | cDNA FLJ10609 fis, clone NT2RP2005276, highly similar to Long-chain-fatty-acid--CoA ligase 3 (EC 6.2.1.3) OS=Homo sapiens PE=2 SV=1 - [B3KMA6_HUMAN] | 10.23 | 6.02 |
| Q14011 | Cold-inducible RNA-binding protein OS=Homo sapiens GN=CIRBP PE=1 SV=1 - [CIRBP_HUMAN] | 10.20 | 26.74 |
| A0A0C4DGS1 | Dolichyl-diphosphooligosaccharide--protein glycosyltransferase 48 kDa subunit OS=Homo sapiens GN=DDOST PE=1 SV=1 - [A0A0C4DGS1_HUMAN] | 9.96 | 2.51 |
| H0YE40 | CD44 antigen (Fragment) OS=Homo sapiens GN=CD44 PE=1 SV=1 - [H0YE40_HUMAN] | 9.78 | 15.85 |
| D6RAN4 | 60S ribosomal protein L9 (Fragment) OS=Homo sapiens GN=RPL9 PE=1 SV=7 - [D6RAN4_HUMAN] | 9.69 | 19.78 |
| Q9BS10 | Similar to ribosomal protein S8 (Fragment) OS=Homo sapiens PE=2 SV=1 - [Q9BS10_HUMAN] | 9.66 | 16.25 |
| D6R991 | Matrin-3 (Fragment) OS=Homo sapiens GN=MATR3 PE=1 SV=1 - [D6R991_HUMAN] | 9.46 | 7.39 |
| Q6J1T2 | Intersectin 1 isoform 7 (Fragment) OS=Homo sapiens GN=ITSN1 PE=2 SV=1 - [Q6J1T2_HUMAN] | 9.36 | 5.70 |
| A0A0J9YVQ7 | ATP-dependent RNA helicase DDX3X (Fragment) OS=Homo sapiens GN=DDX3X PE=1 SV=1 - [A0A0J9YVQ7_HUMAN] | 9.34 | 7.32 |
| A0A024QYX7 | Eukaryotic translation initiation factor 3 subunit C OS=Homo sapiens GN=EIF3S8 PE=3 SV=1 - [A0A024QYX7_HUMAN] | 9.22 | 2.68 |
| Q9BU76 | Multiple myeloma tumor-associated protein 2 OS=Homo sapiens GN=MMTAG2 PE=1 SV=1 - [MMTA2_HUMAN] | 9.21 | 4.94 |
| A4FS09 | Minichromosome maintenance protein 4 (Fragment) | 9.04 | 15.29 |

| | | | |
|---|---|---|---|
| | OS=Homo sapiens GN=MCM4 PE=2 SV=1 - [A4FS09_HUMAN] | | |
| D3DWL9 | Cleavage and polyadenylation specific factor 1, 160kDa, isoform CRA_a OS=Homo sapiens GN=CPSF1 PE=4 SV=1 - [D3DWL9_HUMAN] | 9.02 | 1.25 |
| F2Z388 | 60S ribosomal protein L35 OS=Homo sapiens GN=RPL35 PE=1 SV=1 - [F2Z388_HUMAN] | 8.96 | 10.42 |
| B4DFE6 | cDNA FLJ59861, highly similar to ATP synthase gamma chain, mitochondrial (EC 3.6.3.14) OS=Homo sapiens PE=2 SV=1 - [B4DFE6_HUMAN] | 8.80 | 8.39 |
| H0YNQ3 | Aldehyde dehydrogenase family 1 member A3 OS=Homo sapiens GN=ALDH1A3 PE=1 SV=1 - [H0YNQ3_HUMAN] | 8.77 | 7.80 |
| B4E190 | cDNA FLJ57770, moderately similar to ADP-ribosylation factor 3 OS=Homo sapiens PE=2 SV=1 - [B4E190_HUMAN] | 8.68 | 6.94 |
| B4DM30 | DEAH (Asp-Glu-Ala-His) box polypeptide 38, isoform CRA_d OS=Homo sapiens GN=DHX38 PE=2 SV=1 - [B4DM30_HUMAN] | 8.63 | 2.56 |
| F5GZQ3 | Trifunctional enzyme subunit beta, mitochondrial OS=Homo sapiens GN=HADHB PE=1 SV=1 - [F5GZQ3_HUMAN] | 8.61 | 4.79 |
| A8K9J7 | Histone H2B OS=Homo sapiens PE=2 SV=1 - [A8K9J7_HUMAN] | 7.84 | 11.90 |
| D3DV75 | Adenosine deaminase, RNA-specific, isoform CRA_b OS=Homo sapiens GN=ADAR PE=4 SV=1 - [D3DV75_HUMAN] | 7.80 | 2.03 |
| O43290 | U4/U6.U5 tri-snRNP-associated protein 1 OS=Homo sapiens GN=SART1 PE=1 SV=1 - [SNUT1_HUMAN] | 7.57 | 5.38 |
| B4DRA2 | cDNA FLJ57828, highly similar to Treacle protein (Fragment) OS=Homo sapiens PE=2 SV=1 - [B4DRA2_HUMAN] | 7.44 | 2.71 |
| M1XJE8 | MHC class I antigen (Fragment) OS=Homo sapiens GN=HLA-C PE=3 SV=1 - [M1XJE8_HUMAN] | 7.44 | 8.05 |
| M0R2L9 | 40S ribosomal protein S19 (Fragment) OS=Homo sapiens GN=RPS19 PE=1 SV=1 - [M0R2L9_HUMAN] | 7.31 | 15.49 |
| A0A075B6R9 | Protein IGKV2D-24 (Fragment) OS=Homo sapiens GN=IGKV2D-24 PE=4 SV=1 - [A0A075B6R9_HUMAN] | 7.06 | 10.83 |
| H3BVI7 | Cadherin-1 OS=Homo sapiens GN=CDH1 PE=1 SV=1 - [H3BVI7_HUMAN] | 7.03 | 3.38 |

| | | | |
|---|---|---|---|
| E4W6B6 | RPL27/NME2 fusion protein (Fragment) OS=Homo sapiens GN=RPL27 PE=2 SV=1 - [E4W6B6_HUMAN] | 6.88 | 16.67 |
| B4DDH8 | cDNA FLJ55184, highly similar to Homo sapiens leukocyte receptor cluster (LRC) member 4 (LENG4), mRNA OS=Homo sapiens PE=2 SV=1 - [B4DDH8_HUMAN] | 6.56 | 2.64 |
| Q3SYB5 | SERPINB12 protein OS=Homo sapiens GN=SERPINB12 PE=2 SV=1 - [Q3SYB5_HUMAN] | 6.55 | 8.74 |
| Q14692 | Ribosome biogenesis protein BMS1 homolog OS=Homo sapiens GN=BMS1 PE=1 SV=1 - [BMS1_HUMAN] | 6.50 | 1.09 |
| H0YIZ0 | Serine hydroxymethyltransferase (Fragment) OS=Homo sapiens GN=SHMT2 PE=1 SV=1 - [H0YIZ0_HUMAN] | 6.06 | 4.17 |
| Q15800 | Methylsterol monooxygenase 1 OS=Homo sapiens GN=MSMO1 PE=1 SV=1 - [MSMO1_HUMAN] | 6.02 | 4.78 |
| M0R0Y6 | Heterogeneous nuclear ribonucleoprotein M OS=Homo sapiens GN=HNRNPM PE=1 SV=1 - [M0R0Y6_HUMAN] | 5.97 | 11.11 |
| Q9BSE8 | ZNF607 protein OS=Homo sapiens GN=ZNF607 PE=2 SV=1 - [Q9BSE8_HUMAN] | 5.91 | 6.41 |
| Q8TA90 | Similar to Elongation factor 2b (Fragment) OS=Homo sapiens PE=2 SV=1 - [Q8TA90_HUMAN] | 5.82 | 4.64 |
| Q6DCA8 | BCLAF1 protein (Fragment) OS=Homo sapiens GN=BCLAF1 PE=2 SV=1 - [Q6DCA8_HUMAN] | 5.79 | 3.97 |
| Q9BTC0 | Death-inducer obliterator 1 OS=Homo sapiens GN=DIDO1 PE=1 SV=5 - [DIDO1_HUMAN] | 5.75 | 0.76 |
| B4DZB4 | cDNA FLJ51707, highly similar to Heat-shock protein 105 kDa OS=Homo sapiens PE=2 SV=1 - [B4DZB4_HUMAN] | 5.73 | 2.03 |
| J3KTD9 | Fatty aldehyde dehydrogenase (Fragment) OS=Homo sapiens GN=ALDH3A2 PE=1 SV=1 - [J3KTD9_HUMAN] | 5.71 | 19.35 |
| Q2M243 | Coiled-coil domain-containing protein 27 OS=Homo sapiens GN=CCDC27 PE=2 SV=2 - [CCD27_HUMAN] | 5.56 | 2.29 |
| B4DYP6 | cDNA FLJ57094, highly similar to Probable ATP-dependent RNA helicase DDX47 (EC3.6.1.-) OS=Homo sapiens PE=2 SV=1 - [B4DYP6_HUMAN] | 5.50 | 9.85 |
| F2Z2I2 | 6-phosphofructo-2-kinase/fructose-2,6-bisphosphatas | 5.49 | 2.86 |

| | | | |
|---|---|---|---|
| | e 3 OS=Homo sapiens GN=PFKFB3 PE=1 SV=1 - [F2Z2I2_HUMAN] | | |
| G3V119 | Cell cycle and apoptosis regulator protein 2 OS=Homo sapiens GN=CCAR2 PE=1 SV=1 - [G3V119_HUMAN] | 5.22 | 5.52 |
| B4DIW2 | cDNA FLJ54035, highly similar to Neutral alpha-glucosidase AB OS=Homo sapiens PE=2 SV=1 - [B4DIW2_HUMAN] | 4.63 | 2.53 |
| H3BNP9 | Sulfide:quinone oxidoreductase, mitochondrial (Fragment) OS=Homo sapiens GN=SQRDL PE=1 SV=1 - [H3BNP9_HUMAN] | 4.18 | 11.29 |
| Q8TA86 | Retinitis pigmentosa 9 protein OS=Homo sapiens GN=RP9 PE=1 SV=2 - [RP9_HUMAN] | 3.98 | 4.98 |
| F5GZQ4 | L-lactate dehydrogenase A chain (Fragment) OS=Homo sapiens GN=LDHA PE=1 SV=1 - [F5GZQ4_HUMAN] | 3.92 | 20.83 |
| Q5VVC9 | 60S ribosomal protein L11 (Fragment) OS=Homo sapiens GN=RPL11 PE=1 SV=1 - [Q5VVC9_HUMAN] | 3.91 | 10.69 |
| B3KN82 | cDNA FLJ13913 fis, clone Y79AA1000231, highly similar to Nucleolar protein NOP5 OS=Homo sapiens PE=2 SV=1 - [B3KN82_HUMAN] | 3.90 | 2.79 |
| P01275 | Glucagon OS=Homo sapiens GN=GCG PE=1 SV=3 - [GLUC_HUMAN] | 3.82 | 11.11 |
| A1L1B8 | FSIP2 protein (Fragment) OS=Homo sapiens GN=FSIP2 PE=2 SV=1 - [A1L1B8_HUMAN] | 3.79 | 13.85 |
| B3KY11 | cDNA FLJ46571 fis, clone THYMU3041428, highly similar to Probable ATP-dependent RNA helicase DDX23 (EC 3.6.1.-) OS=Homo sapiens PE=2 SV=1 - [B3KY11_HUMAN] | 3.70 | 2.00 |
| F5H7Y1 | T-complex protein 1 subunit alpha (Fragment) OS=Homo sapiens GN=TCP1 PE=1 SV=1 - [F5H7Y1_HUMAN] | 3.68 | 25.42 |
| Q5H9P4 | Putative uncharacterized protein DKFZp686M19106 (Fragment) OS=Homo sapiens GN=DKFZp686M19106 PE=4 SV=1 - [Q5H9P4_HUMAN] | 3.67 | 1.41 |
| Q1LZN2 | NOMO3 protein (Fragment) OS=Homo sapiens GN=NOMO3 PE=2 SV=1 - [Q1LZN2_HUMAN] | 3.64 | 1.36 |
| P11766 | Alcohol dehydrogenase class-3 OS=Homo sapiens GN=ADH5 PE=1 SV=4 - [ADHX_HUMAN] | 3.61 | 4.28 |
| E0WBQ9 | MHC class I antigen (Fragment) OS=Homo sapiens GN=HLA-C PE=3 SV=1 - [E0WBQ9_HUMAN] | 3.60 | 11.05 |
| Q59FN3 | Acyl-Coenzyme A dehydrogenase family, member 9 | 3.58 | 5.47 |

| | | | |
|---|---|---|---|
| | variant (Fragment) OS=Homo sapiens PE=2 SV=1 - [Q59FN3_HUMAN] | | |
| A0A0U1RQV5 | Uncharacterized protein (Fragment) OS=Homo sapiens PE=4 SV=1 - [A0A0U1RQV5_HUMAN] | 3.56 | 21.82 |
| P02814 | Submaxillary gland androgen-regulated protein 3B OS=Homo sapiens GN=SMR3B PE=1 SV=2 - [SMR3B_HUMAN] | 3.55 | 26.58 |
| A0A0C4DH52 | Constitutive coactivator of PPAR-gamma-like protein 1 (Fragment) OS=Homo sapiens GN=FAM120A PE=1 SV=1 - [A0A0C4DH52_HUMAN] | 3.53 | 3.43 |
| A0A024R326 | Ribosomal protein L29, isoform CRA_a OS=Homo sapiens GN=RPL29 PE=4 SV=1 - [A0A024R326_HUMAN] | 3.51 | 9.55 |
| M0QXM4 | Amino acid transporter OS=Homo sapiens GN=SLC1A5 PE=1 SV=1 - [M0QXM4_HUMAN] | 3.51 | 5.48 |
| P0C0S5 | Histone H2A.Z OS=Homo sapiens GN=H2AFZ PE=1 SV=2 - [H2AZ_HUMAN] | 3.48 | 10.94 |
| E5RFH5 | Set1/Ash2 histone methyltransferase complex subunit ASH2 (Fragment) OS=Homo sapiens GN=ASH2L PE=1 SV=1 - [E5RFH5_HUMAN] | 3.47 | 12.94 |
| S4R456 | 40S ribosomal protein S15 (Fragment) OS=Homo sapiens GN=RPS15 PE=1 SV=1 - [S4R456_HUMAN] | 3.43 | 27.94 |
| B4DKX6 | cDNA FLJ53584, highly similar to Desmoplakin (Fragment) OS=Homo sapiens PE=2 SV=1 - [B4DKX6_HUMAN] | 3.43 | 2.20 |
| H0Y8X1 | Succinate dehydrogenase [ubiquinone] flavoprotein subunit, mitochondrial (Fragment) OS=Homo sapiens GN=SDHA PE=1 SV=1 - [H0Y8X1_HUMAN] | 3.43 | 11.11 |
| Q6AZY7 | Scavenger receptor class A member 3 OS=Homo sapiens GN=SCARA3 PE=1 SV=1 - [SCAR3_HUMAN] | 3.40 | 4.46 |
| A0A0S2Z4X1 | RNA binding motif protein 10 isoform 1 (Fragment) OS=Homo sapiens GN=RBM10 PE=2 SV=1 - [A0A0S2Z4X1_HUMAN] | 3.38 | 1.88 |
| P01833 | Polymeric immunoglobulin receptor OS=Homo sapiens GN=PIGR PE=1 SV=4 - [PIGR_HUMAN] | 3.36 | 2.62 |
| O60762 | Dolichol-phosphate mannosyltransferase subunit 1 OS=Homo sapiens GN=DPM1 PE=1 SV=1 - [DPM1_HUMAN] | 3.36 | 6.15 |
| Q9UG16 | Putative uncharacterized protein DKFZp564P0562 (Fragment) OS=Homo sapiens GN=DKFZp564P0562 PE=2 SV=1 - [Q9UG16_HUMAN] | 3.35 | 0.91 |

| | | | |
|---|---|---|---|
| E9PLX0 | Calpain-1 catalytic subunit (Fragment) OS=Homo sapiens GN=CAPN1 PE=1 SV=1 - [E9PLX0_HUMAN] | 3.34 | 14.75 |
| A0A075BTL2 | Endoplasmic reticulum aminopeptidase 1 delta-Exon-11 isoform OS=Homo sapiens GN=ERAP1 PE=2 SV=1 - [A0A075BTL2_HUMAN] | 3.21 | 2.37 |
| Q9HAP1 | Valosin-containing protein (Fragment) OS=Homo sapiens GN=VCP PE=2 SV=1 - [Q9HAP1_HUMAN] | 3.19 | 5.54 |
| Q86WX3 | Active regulator of SIRT1 OS=Homo sapiens GN=RPS19BP1 PE=1 SV=1 - [AROS_HUMAN] | 3.16 | 9.56 |
| J3KTL8 | Structural maintenance of chromosomes flexible hinge domain-containing protein 1 (Fragment) OS=Homo sapiens GN=SMCHD1 PE=1 SV=1 - [J3KTL8_HUMAN] | 3.16 | 1.08 |
| A1XP52 | Catecholamine-regulated protein 40 OS=Homo sapiens PE=2 SV=1 - [A1XP52_HUMAN] | 3.10 | 3.43 |
| A0A0S2Z4Q6 | Hydroxysteroid dehydrogenase 4 isoform 4 OS=Homo sapiens GN=HSD17B4 PE=2 SV=1 - [A0A0S2Z4Q6_HUMAN] | 3.08 | 16.47 |
| Q1RMG2 | Adenosylhomocysteinase OS=Homo sapiens GN=AHCY PE=2 SV=1 - [Q1RMG2_HUMAN] | 3.06 | 3.92 |
| H7C1I9 | Microtubule-associated serine/threonine-protein kinase 4 (Fragment) OS=Homo sapiens GN=MAST4 PE=1 SV=1 - [H7C1I9_HUMAN] | 3.05 | 0.95 |
| K7EJT5 | 60S ribosomal protein L22 (Fragment) OS=Homo sapiens GN=RPL22 PE=1 SV=1 - [K7EJT5_HUMAN] | 3.04 | 23.40 |
| Q9BVI4 | Nucleolar complex protein 4 homolog OS=Homo sapiens GN=NOC4L PE=1 SV=1 - [NOC4L_HUMAN] | 3.01 | 1.74 |
| H7C5W9 | Sarcoplasmic/endoplasmic reticulum calcium ATPase 2 (Fragment) OS=Homo sapiens GN=ATP2A2 PE=1 SV=1 - [H7C5W9_HUMAN] | 3.00 | 1.39 |
| A0A0A0MRQ5 | Peroxiredoxin-1 OS=Homo sapiens GN=PRDX1 PE=1 SV=1 - [A0A0A0MRQ5_HUMAN] | 3.00 | 11.34 |
| H0Y8L8 | Probable ATP-dependent RNA helicase DDX41 (Fragment) OS=Homo sapiens GN=DDX41 PE=1 SV=1 - [H0Y8L8_HUMAN] | 3.00 | 6.36 |
| E9PMJ3 | Ribonuclease inhibitor (Fragment) OS=Homo sapiens GN=RNH1 PE=1 SV=1 - [E9PMJ3_HUMAN] | 2.99 | 7.94 |
| F8VV32 | Lysozyme C OS=Homo sapiens GN=LYZ PE=1 SV=1 - [F8VV32_HUMAN] | 2.98 | 11.54 |

| | | | |
|---|---|---|---|
| B4DN27 | cDNA FLJ57057, highly similar to Small subunit processome component 20 homolog OS=Homo sapiens PE=2 SV=1 - [B4DN27_HUMAN] | 2.97 | 2.40 |
| Q03252 | Lamin-B2 OS=Homo sapiens GN=LMNB2 PE=1 SV=4 - [LMNB2_HUMAN] | 2.97 | 1.61 |
| B3KNN7 | cDNA FLJ30049 fis, clone ADRGL1000033, highly similar to 26S proteasome non-ATPase regulatory subunit 3 OS=Homo sapiens PE=2 SV=1 - [B3KNN7_HUMAN] | 2.96 | 3.01 |
| B4DNS2 | cDNA FLJ51602, highly similar to Interferon-induced guanylate-binding protein 1 OS=Homo sapiens PE=2 SV=1 - [B4DNS2_HUMAN] | 2.92 | 3.36 |
| Q6P0M4 | IARS protein OS=Homo sapiens GN=IARS PE=2 SV=1 - [Q6P0M4_HUMAN] | 2.92 | 0.95 |
| B4DZH8 | cDNA FLJ50311, highly similar to Tumor necrosis factor, alpha-induced protein 2 OS=Homo sapiens PE=2 SV=1 - [B4DZH8_HUMAN] | 2.92 | 3.41 |
| B4E0F7 | cDNA FLJ61512 OS=Homo sapiens PE=2 SV=1 - [B4E0F7_HUMAN] | 2.91 | 4.58 |
| M0QZK8 | Uncharacterized protein OS=Homo sapiens PE=4 SV=1 - [M0QZK8_HUMAN] | 2.91 | 9.71 |
| A0A0A0MQZ6 | Ribosome biogenesis protein NSA2 homolog OS=Homo sapiens GN=NSA2 PE=1 SV=1 - [A0A0A0MQZ6_HUMAN] | 2.89 | 6.88 |
| A0A024QZT8 | Serpin peptidase inhibitor, clade B (Ovalbumin), member 6, isoform CRA_b OS=Homo sapiens GN=SERPINB6 PE=3 SV=1 - [A0A024QZT8_HUMAN] | 2.86 | 7.51 |
| B4DFQ0 | cDNA FLJ60239, highly similar to Proto-oncogene tyrosine-protein kinase MER (EC 2.7.10.1) OS=Homo sapiens PE=2 SV=1 - [B4DFQ0_HUMAN] | 2.86 | 4.52 |
| P62861 | 40S ribosomal protein S30 OS=Homo sapiens GN=FAU PE=1 SV=1 - [RS30_HUMAN] | 2.84 | 18.64 |
| Q15365 | Poly(rC)-binding protein 1 OS=Homo sapiens GN=PCBP1 PE=1 SV=2 - [PCBP1_HUMAN] | 2.84 | 3.65 |
| A0A0C4DGH5 | Cullin-associated NEDD8-dissociated protein 1 (Fragment) OS=Homo sapiens GN=CAND1 PE=1 SV=1 - [A0A0C4DGH5_HUMAN] | 2.83 | 1.43 |
| Q4JFL9 | Protein S100 (Fragment) OS=Homo sapiens GN=FLG PE=2 SV=1 - [Q4JFL9_HUMAN] | 2.81 | 10.87 |
| B4DWX8 | cDNA FLJ52669, highly similar to Regulator of G-protein signaling 9 OS=Homo sapiens PE=2 SV=1 | 2.81 | 2.40 |

| | | | |
|---|---|---|---|
| | - [B4DWX8_HUMAN] | | |
| A7E2T1 | Uncharacterized protein (Fragment) OS=Homo sapiens PE=2 SV=1 - [A7E2T1_HUMAN] | 2.80 | 13.39 |
| Q49AJ9 | RPL3 protein OS=Homo sapiens GN=RPL3 PE=2 SV=1 - [Q49AJ9_HUMAN] | 2.79 | 4.78 |
| A0A087X2D0 | Serine/arginine-rich-splicing factor 3 OS=Homo sapiens GN=SRSF3 PE=1 SV=1 - [A0A087X2D0_HUMAN] | 2.78 | 14.74 |
| B3KSC3 | cDNA FLJ35987 fis, clone TESTI2014269, highly similar to D-3-phosphoglycerate dehydrogenase (EC 1.1.1.95) OS=Homo sapiens PE=2 SV=1 - [B3KSC3_HUMAN] | 2.78 | 3.01 |
| D3DPU8 | Collagen, type IX, alpha 2, isoform CRA_a OS=Homo sapiens GN=COL9A2 PE=4 SV=1 - [D3DPU8_HUMAN] | 2.78 | 2.35 |
| B4E1D4 | cDNA FLJ54399, highly similar to Golgin subfamily A member 5 OS=Homo sapiens PE=2 SV=1 - [B4E1D4_HUMAN] | 2.77 | 3.13 |
| Q4ZG72 | Putative uncharacterized protein DDX18 (Fragment) OS=Homo sapiens GN=DDX18 PE=3 SV=1 - [Q4ZG72_HUMAN] | 2.76 | 1.83 |
| D6RHJ3 | Calnexin (Fragment) OS=Homo sapiens GN=CANX PE=1 SV=7 - [D6RHJ3_HUMAN] | 2.76 | 20.25 |
| H3BUH7 | Fructose-bisphosphate aldolase A (Fragment) OS=Homo sapiens GN=ALDOA PE=1 SV=1 - [H3BUH7_HUMAN] | 2.76 | 9.03 |
| Q9Y657 | Spindlin-1 OS=Homo sapiens GN=SPIN1 PE=1 SV=3 - [SPIN1_HUMAN] | 2.76 | 3.82 |
| P15515 | Histatin-1 OS=Homo sapiens GN=HTN1 PE=1 SV=2 - [HIS1_HUMAN] | 2.75 | 28.07 |
| F8W0G4 | Poly(rC)-binding protein 2 (Fragment) OS=Homo sapiens GN=PCBP2 PE=1 SV=1 - [F8W0G4_HUMAN] | 2.75 | 8.23 |
| H0Y920 | Mastermind-like protein 3 (Fragment) OS=Homo sapiens GN=MAML3 PE=1 SV=1 - [H0Y920_HUMAN] | 2.75 | 8.06 |
| H0YFC6 | GTP-binding nuclear protein Ran (Fragment) OS=Homo sapiens GN=RAN PE=1 SV=1 - [H0YFC6_HUMAN] | 2.75 | 10.68 |
| O15327 | Type II inositol 3,4-bisphosphate 4-phosphatase OS=Homo sapiens GN=INPP4B PE=2 SV=4 - [INP4B_HUMAN] | 2.72 | 1.41 |
| B3KQH1 | cDNA FLJ90452 fis, clone NT2RP3001475, highly similar to Splicing factor 3B subunit 3 OS=Homo | 2.71 | 1.56 |

| | | | |
|---|---|---|---|
| | sapiens PE=2 SV=1 - [B3KQH1_HUMAN] | | |
| F6UXX1 | Heterogeneous nuclear ribonucleoprotein Q (Fragment) OS=Homo sapiens GN=SYNCRIP PE=1 SV=1 - [F6UXX1_HUMAN] | 2.71 | 7.03 |
| P0C1S8 | Wee1-like protein kinase 2 OS=Homo sapiens GN=WEE2 PE=2 SV=2 - [WEE2_HUMAN] | 2.70 | 3.17 |
| B4DT32 | cDNA FLJ54383, highly similar to Valyl-tRNA synthetase (EC 6.1.1.9) OS=Homo sapiens PE=2 SV=1 - [B4DT32_HUMAN] | 2.70 | 1.78 |
| Q7KZP0 | Catechol-O-methyltransferase OS=Homo sapiens PE=2 SV=1 - [Q7KZP0_HUMAN] | 2.68 | 30.95 |
| B4DSR0 | cDNA FLJ60080, highly similar to 130 kDa leucine-rich protein (LRP 130) (Fragment) OS=Homo sapiens PE=2 SV=1 - [B4DSR0_HUMAN] | 2.68 | 1.01 |
| B4DTH5 | cDNA FLJ55592, weakly similar to Sel-1 homolog OS=Homo sapiens PE=2 SV=1 - [B4DTH5_HUMAN] | 2.67 | 3.53 |

**Supplementary table 2. The information of HPV 11E7 interacted proteins in stable expression of HPV 11E7 HaCaT cells after transfection with poly(dA:dT).**

| Accession | Description | Score | Coverage |
|---|---|---|---|
| P06400 | Retinoblastoma-associated protein OS=Homo sapiens GN=RB1 PE=1 SV=2 - [RB_HUMAN] | 297.41 | 27.48 |
| P28749 | Retinoblastoma-like protein 1 OS=Homo sapiens GN=RBL1 PE=1 SV=3 - [RBL1_HUMAN] | 234.59 | 19.01 |
| B7ZMG8 | Uncharacterized protein OS=Homo sapiens PE=2 SV=1 - [B7ZMG8_HUMAN] | 204.79 | 15.66 |
| P19474 | E3 ubiquitin-protein ligase TRIM21 OS=Homo sapiens GN=TRIM21 PE=1 SV=1 - [RO52_HUMAN] | 201.07 | 18.53 |
| C9JIR6 | Protein phosphatase 1B (Fragment) OS=Homo sapiens GN=PPM1B PE=1 SV=1 - [C9JIR6_HUMAN] | 123.36 | 16.40 |

| | | | |
|---|---|---|---|
| Q8WUW7 | Pyruvate kinase (Fragment) OS=Homo sapiens GN=PKM2 PE=2 SV=2 - [Q8WUW7_HUMAN] | 78.43 | 21.28 |
| B0QYK0 | RNA-binding protein EWS OS=Homo sapiens GN=EWSR1 PE=1 SV=1 - [B0QYK0_HUMAN] | 71.82 | 9.39 |
| B7Z3V1 | Sodium/potassium-transporting ATPase subunit alpha (Fragment) OS=Homo sapiens PE=2 SV=1 - [B7Z3V1_HUMAN] | 58.31 | 9.22 |
| B3KML9 | cDNA FLJ11352 fis, clone HEMBA1000020, highly similar to Tubulin beta-2C chain OS=Homo sapiens PE=2 SV=1 - [B3KML9_HUMAN] | 56.57 | 19.14 |
| B4DW52 | cDNA FLJ55253, highly similar to Actin, cytoplasmic 1 OS=Homo sapiens PE=2 SV=1 - [B4DW52_HUMAN] | 55.52 | 22.19 |
| Q96RE1 | Translation elongation factor 1 alpha 1-like 14 OS=Homo sapiens GN=EEF1A1L14 PE=2 SV=1 - [Q96RE1_HUMAN] | 53.70 | 14.82 |
| Q0QET7 | Glyceraldehyde-3-phosphate dehydrogenase (Fragment) OS=Homo sapiens GN=GAPDH PE=2 SV=1 - [Q0QET7_HUMAN] | 52.15 | 26.09 |
| V9HWC6 | Peptidyl-prolyl cis-trans isomerase OS=Homo sapiens GN=HEL-S-39 PE=2 SV=1 - [V9HWC6_HUMAN] | 51.14 | 12.50 |
| B2RDD7 | Protein arginine N-methyltransferase 5 OS=Homo sapiens PE=2 SV=1 - [B2RDD7_HUMAN] | 48.59 | 12.24 |
| Q9NXV2 | BTB/POZ domain-containing protein KCTD5 OS=Homo sapiens GN=KCTD5 PE=1 SV=1 - [KCTD5_HUMAN] | 47.96 | 26.50 |
| Q9BV28 | TUBB3 protein (Fragment) OS=Homo sapiens GN=TUBB3 PE=2 SV=2 - [Q9BV28_HUMAN] | 47.75 | 12.81 |

| | | | |
|---|---|---|---|
| A8K6H6 | cDNA FLJ76875, highly similar to Homo sapiens protein tyrosine phosphatase, non-receptor type 14 (PTPN14), mRNA OS=Homo sapiens PE=2 SV=1 - [A8K6H6_HUMAN] | 40.18 | 7.58 |
| Q6P1L8 | 39S ribosomal protein L14, mitochondrial OS=Homo sapiens GN=MRPL14 PE=1 SV=1 - [RM14_HUMAN] | 39.09 | 20.00 |
| Q8WUT1 | POLDIP3 protein OS=Homo sapiens GN=POLDIP3 PE=1 SV=1 - [Q8WUT1_HUMAN] | 38.89 | 15.72 |
| P27816 | Microtubule-associated protein 4 OS=Homo sapiens GN=MAP4 PE=1 SV=3 - [MAP4_HUMAN] | 36.11 | 3.91 |
| E9PKE3 | Heat shock cognate 71 kDa protein OS=Homo sapiens GN=HSPA8 PE=1 SV=1 - [E9PKE3_HUMAN] | 34.10 | 10.69 |
| A0A0U1RQF0 | Fatty acid synthase OS=Homo sapiens GN=FASN PE=4 SV=1 - [A0A0U1RQF0_HUMAN] | 33.87 | 0.96 |
| B3KU16 | cDNA FLJ39066 fis, clone NT2RP7014743, highly similar to HETEROGENEOUS NUCLEAR RIBONUCLEOPROTEIN K OS=Homo sapiens PE=2 SV=1 - [B3KU16_HUMAN] | 33.76 | 18.83 |
| F8VSC5 | SCY1-like protein 2 (Fragment) OS=Homo sapiens GN=SCYL2 PE=1 SV=1 - [F8VSC5_HUMAN] | 29.76 | 8.22 |
| Q969I0 | KRT8 protein (Fragment) OS=Homo sapiens GN=KRT8 PE=2 SV=2 - [Q969I0_HUMAN] | 28.98 | 17.26 |
| B4DSZ9 | E3 ubiquitin-protein ligase TTC3 OS=Homo sapiens GN=TTC3 PE=1 SV=1 - [B4DSZ9_HUMAN] | 28.88 | 2.61 |
| P30050 | 60S ribosomal protein L12 OS=Homo sapiens GN=RPL12 PE=1 SV=1 - [RL12_HUMAN] | 28.12 | 19.39 |

| | | | |
|---|---|---|---|
| Q99714 | 3-hydroxyacyl-CoA dehydrogenase type-2 OS=Homo sapiens GN=HSD17B10 PE=1 SV=3 - [HCD2_HUMAN] | 27.92 | 19.92 |
| E7EN95 | Filamin-B OS=Homo sapiens GN=FLNB PE=1 SV=1 - [E7EN95_HUMAN] | 27.51 | 2.16 |
| Q5JYR4 | Dolichyl-diphosphooligosaccharide--protein glycosyltransferase subunit 2 (Fragment) OS=Homo sapiens GN=RPN2 PE=1 SV=7 - [Q5JYR4_HUMAN] | 26.82 | 17.37 |
| A8K9J7 | Histone H2B OS=Homo sapiens PE=2 SV=1 - [A8K9J7_HUMAN] | 26.77 | 11.90 |
| F2Z2S8 | 40S ribosomal protein S3 OS=Homo sapiens GN=RPS3 PE=1 SV=1 - [F2Z2S8_HUMAN] | 26.43 | 29.06 |
| C9JLM5 | Serpin B5 (Fragment) OS=Homo sapiens GN=SERPINB5 PE=1 SV=1 - [C9JLM5_HUMAN] | 26.09 | 11.63 |
| Q13707 | ACTA2 protein (Fragment) OS=Homo sapiens GN=ACTA2 PE=3 SV=1 - [Q13707_HUMAN] | 24.16 | 9.70 |
| Q96P47 | Arf-GAP with GTPase, ANK repeat and PH domain-containing protein 3 OS=Homo sapiens GN=AGAP3 PE=1 SV=2 - [AGAP3_HUMAN] | 23.44 | 4.69 |
| F5H837 | Retinoblastoma-like protein 2 (Fragment) OS=Homo sapiens GN=RBL2 PE=1 SV=2 - [F5H837_HUMAN] | 23.20 | 4.38 |
| Q2VPJ6 | HSP90AA1 protein (Fragment) OS=Homo sapiens GN=HSP90AA1 PE=1 SV=1 - [Q2VPJ6_HUMAN] | 23.15 | 8.21 |
| A0A024R599 | Solute carrier family 3 (Activators of dibasic and neutral amino acid transport), member 2, isoform | 22.94 | 7.29 |

| | | | |
|---|---|---|---|
| | CRA_e OS=Homo sapiens GN=SLC3A2 PE=4 SV=1 - [A0A024R599_HUMAN] | | |
| B4DUP0 | cDNA FLJ59433, highly similar to Elongation factor 1-gamma OS=Homo sapiens PE=2 SV=1 - [B4DUP0_HUMAN] | 22.42 | 6.80 |
| Q9UHS8 | PRO1975 OS=Homo sapiens PE=2 SV=1 - [Q9UHS8_HUMAN] | 22.03 | 7.12 |
| Q9Y2W1 | Thyroid hormone receptor-associated protein 3 OS=Homo sapiens GN=THRAP3 PE=1 SV=2 - [TR150_HUMAN] | 20.27 | 4.08 |
| Q86V81 | THO complex subunit 4 OS=Homo sapiens GN=ALYREF PE=1 SV=3 - [THOC4_HUMAN] | 18.38 | 7.00 |
| Q8IYY2 | KCTD2 protein (Fragment) OS=Homo sapiens GN=KCTD2 PE=2 SV=1 - [Q8IYY2_HUMAN] | 18.02 | 11.43 |
| Q02413 | Desmoglein-1 OS=Homo sapiens GN=DSG1 PE=1 SV=2 - [DSG1_HUMAN] | 17.34 | 1.53 |
| B4DN39 | cDNA FLJ53065, highly similar to T-complex protein 1 subunit zeta OS=Homo sapiens PE=2 SV=1 - [B4DN39_HUMAN] | 16.60 | 4.11 |
| F6IB50 | MHC class I antigen (Fragment) OS=Homo sapiens GN=HLA-A PE=4 SV=1 - [F6IB50_HUMAN] | 16.25 | 14.13 |
| O15327 | Type II inositol 3,4-bisphosphate 4-phosphatase OS=Homo sapiens GN=INPP4B PE=2 SV=4 - [INP4B_HUMAN] | 16.12 | 1.41 |
| B4DH39 | cDNA FLJ57028, highly similar to Long-chain-fatty-acid--CoA ligase 4 (EC 6.2.1.3) OS=Homo sapiens PE=2 SV=1 - [B4DH39_HUMAN] | 15.42 | 5.08 |
| Q6J1T2 | Intersectin 1 isoform 7 (Fragment) OS=Homo | 15.09 | 5.70 |

| | | | |
|---|---|---|---|
| | sapiens GN=ITSN1 PE=2 SV=1 - [Q6J1T2_HUMAN] | | |
| B4DWX8 | cDNA FLJ52669, highly similar to Regulator of G-protein signaling 9 OS=Homo sapiens PE=2 SV=1 - [B4DWX8_HUMAN] | 14.99 | 2.40 |
| F8W0P7 | ATP synthase subunit beta, mitochondrial (Fragment) OS=Homo sapiens GN=ATP5B PE=1 SV=2 - [F8W0P7_HUMAN] | 13.50 | 5.19 |
| F8VV32 | Lysozyme C OS=Homo sapiens GN=LYZ PE=1 SV=1 - [F8VV32_HUMAN] | 13.35 | 11.54 |
| B4DRH6 | cDNA FLJ54509, highly similar to Trifunctional enzyme subunit alpha, mitochondrial OS=Homo sapiens PE=2 SV=1 - [B4DRH6_HUMAN] | 12.94 | 5.02 |
| Q9BS10 | Similar to ribosomal protein S8 (Fragment) OS=Homo sapiens PE=2 SV=1 - [Q9BS10_HUMAN] | 12.91 | 16.25 |
| B4DHT9 | cDNA FLJ58812, highly similar to Endoplasmin (Heat shock protein 90kDa beta member 1) OS=Homo sapiens PE=2 SV=1 - [B4DHT9_HUMAN] | 12.05 | 2.99 |
| B3KM90 | cDNA FLJ10529 fis, clone NT2RP2000965, highly similar to Targeting protein for Xklp2 OS=Homo sapiens PE=2 SV=1 - [B3KM90_HUMAN] | 12.05 | 4.13 |
| Q9UNW1 | Multiple inositol polyphosphate phosphatase 1 OS=Homo sapiens GN=MINPP1 PE=1 SV=1 - [MINP1_HUMAN] | 11.88 | 4.52 |
| J3KTD9 | Fatty aldehyde dehydrogenase (Fragment) OS=Homo sapiens GN=ALDH3A2 PE=1 SV=1 - [J3KTD9_HUMAN] | 11.78 | 19.35 |

| | | | |
|---|---|---|---|
| Q9P172 | PRO2281 OS=Homo sapiens PE=2 SV=1 - [Q9P172_HUMAN] | 11.47 | 9.73 |
| H0Y9R4 | 60S ribosomal protein L9 (Fragment) OS=Homo sapiens GN=RPL9 PE=1 SV=1 - [H0Y9R4_HUMAN] | 10.37 | 29.67 |
| A0A087WWP1 | Ras GTPase-activating-like protein IQGAP1 (Fragment) OS=Homo sapiens GN=IQGAP1 PE=1 SV=1 - [A0A087WWP1_HUMAN] | 9.74 | 14.95 |
| Q5JSD2 | Voltage-dependent anion-selective channel protein 2 (Fragment) OS=Homo sapiens GN=VDAC2 PE=1 SV=7 - [Q5JSD2_HUMAN] | 9.74 | 6.67 |
| Q7Z5X3 | EIF3L protein OS=Homo sapiens GN=EIF3L PE=2 SV=2 - [Q7Z5X3_HUMAN] | 9.27 | 8.51 |
| P62081 | 40S ribosomal protein S7 OS=Homo sapiens GN=RPS7 PE=1 SV=1 - [RS7_HUMAN] | 8.98 | 11.86 |
| P46783 | 40S ribosomal protein S10 OS=Homo sapiens GN=RPS10 PE=1 SV=1 - [RS10_HUMAN] | 8.63 | 8.48 |
| Q68GS6 | Epidermal growth factor receptor (Fragment) OS=Homo sapiens GN=EGFR PE=2 SV=1 - [Q68GS6_HUMAN] | 8.60 | 3.57 |
| B4DRA2 | cDNA FLJ57828, highly similar to Treacle protein (Fragment) OS=Homo sapiens PE=2 SV=1 - [B4DRA2_HUMAN] | 8.38 | 1.08 |
| Q86UM1 | Putative uncharacterized protein EIF3S9 (Fragment) OS=Homo sapiens GN=EIF3S9 PE=4 SV=1 - [Q86UM1_HUMAN] | 7.61 | 15.06 |
| Q86Y46 | Keratin, type II cytoskeletal 73 OS=Homo sapiens GN=KRT73 PE=1 SV=1 - [K2C73_HUMAN] | 7.54 | 4.44 |
| H0YE40 | CD44 antigen (Fragment) OS=Homo sapiens | 7.33 | 35.37 |

| | | | |
|---|---|---|---|
| | GN=CD44 PE=1 SV=1 - [H0YE40_HUMAN] | | |
| J3QL15 | Ribosomal protein L19 (Fragment) OS=Homo sapiens GN=RPL19 PE=1 SV=1 - [J3QL15_HUMAN] | 7.21 | 13.28 |
| A0A075B6R9 | Protein IGKV2D-24 (Fragment) OS=Homo sapiens GN=IGKV2D-24 PE=4 SV=1 - [A0A075B6R9_HUMAN] | 6.66 | 10.83 |
| A0A024R409 | Microtubule-associated protein OS=Homo sapiens GN=MAP2 PE=4 SV=1 - [A0A024R409_HUMAN] | 6.54 | 5.10 |
| B2RAM6 | Kinesin-like protein OS=Homo sapiens PE=2 SV=1 - [B2RAM6_HUMAN] | 5.90 | 2.27 |
| B4DVK5 | cDNA FLJ54759, highly similar to DNA replication licensing factor MCM5 OS=Homo sapiens PE=2 SV=1 - [B4DVK5_HUMAN] | 5.76 | 3.16 |
| F8W0G4 | Poly(rC)-binding protein 2 (Fragment) OS=Homo sapiens GN=PCBP2 PE=1 SV=1 - [F8W0G4_HUMAN] | 5.73 | 8.23 |
| Q96J17 | NOLC1 protein OS=Homo sapiens GN=NOLC1 PE=2 SV=1 - [Q96J17_HUMAN] | 5.69 | 2.63 |
| A8K525 | cDNA FLJ76817, highly similar to Homo sapiens non-POU domain containing, octamer-binding (NONO), mRNA OS=Homo sapiens PE=2 SV=1 - [A8K525_HUMAN] | 4.83 | 4.67 |
| F5H2D2 | Prohibitin-2 OS=Homo sapiens GN=PHB2 PE=1 SV=1 - [F5H2D2_HUMAN] | 4.46 | 19.54 |
| B4DJP0 | cDNA FLJ60601, highly similar to Methylosome protein 50 OS=Homo sapiens PE=2 SV=1 - [B4DJP0_HUMAN] | 4.44 | 13.39 |

| | | | |
|---|---|---|---|
| A0A0S2Z415 | Heat shock 60kDa protein 1 isoform 4 OS=Homo sapiens GN=HSPD1 PE=2 SV=1 - [A0A0S2Z415_HUMAN] | 4.37 | 14.79 |
| A0A024QYX7 | Eukaryotic translation initiation factor 3 subunit C OS=Homo sapiens GN=EIF3S8 PE=3 SV=1 - [A0A024QYX7_HUMAN] | 4.35 | 2.68 |
| Q86W20 | Protease serine 1 (Fragment) OS=Homo sapiens GN=PRSS1 PE=3 SV=1 - [Q86W20_HUMAN] | 4.34 | 23.81 |
| Q53GM6 | U5 snRNP-specific protein variant (Fragment) OS=Homo sapiens PE=2 SV=1 - [Q53GM6_HUMAN] | 4.25 | 3.52 |
| E9PQI8 | U4/U6.U5 tri-snRNP-associated protein 1 OS=Homo sapiens GN=SART1 PE=1 SV=1 - [E9PQI8_HUMAN] | 3.80 | 12.20 |
| A0A087WXU5 | Nucleolar protein of 40 kDa OS=Homo sapiens GN=ZCCHC17 PE=1 SV=1 - [A0A087WXU5_HUMAN] | 3.72 | 9.59 |
| Q6ZNL4 | FLJ00279 protein (Fragment) OS=Homo sapiens GN=FLJ00279 PE=2 SV=1 - [Q6ZNL4_HUMAN] | 3.67 | 2.31 |
| B3KUD7 | DNA helicase OS=Homo sapiens PE=2 SV=1 - [B3KUD7_HUMAN] | 3.54 | 2.12 |
| H0Y8X1 | Succinate dehydrogenase [ubiquinone] flavoprotein subunit, mitochondrial (Fragment) OS=Homo sapiens GN=SDHA PE=1 SV=1 - [H0Y8X1_HUMAN] | 3.37 | 11.11 |
| E9PKC4 | Plakophilin-3 (Fragment) OS=Homo sapiens GN=PKP3 PE=1 SV=1 - [E9PKC4_HUMAN] | 3.36 | 7.61 |
| H0YMU6 | Phosphoenolpyruvate carboxykinase [GTP], mitochondrial (Fragment) OS=Homo sapiens | 3.31 | 14.95 |

| | | | |
|---|---|---|---|
| | GN=PCK2 PE=1 SV=1 - [H0YMU6_HUMAN] | | |
| A0A0S2Z4X1 | RNA binding motif protein 10 isoform 1 (Fragment) OS=Homo sapiens GN=RBM10 PE=2 SV=1 - [A0A0S2Z4X1_HUMAN] | 3.28 | 1.88 |
| S4R3N9 | Sideroflexin-3 OS=Homo sapiens GN=SFXN3 PE=1 SV=1 - [S4R3N9_HUMAN] | 3.26 | 7.34 |
| B4DNS2 | cDNA FLJ51602, highly similar to Interferon-induced guanylate-binding protein 1 OS=Homo sapiens PE=2 SV=1 - [B4DNS2_HUMAN] | 3.23 | 3.36 |
| Q53T09 | Putative uncharacterized protein XRCC5 (Fragment) OS=Homo sapiens GN=XRCC5 PE=4 SV=1 - [Q53T09_HUMAN] | 3.22 | 2.11 |
| B5MD38 | Trifunctional enzyme subunit beta, mitochondrial OS=Homo sapiens GN=HADHB PE=1 SV=1 - [B5MD38_HUMAN] | 3.19 | 3.70 |
| R4GN64 | Calcium-binding mitochondrial carrier protein Aralar2 OS=Homo sapiens GN=SLC25A13 PE=1 SV=1 - [R4GN64_HUMAN] | 3.11 | 17.81 |
| F2Z2I2 | 6-phosphofructo-2-kinase/fructose-2,6-bisphosphata se 3 OS=Homo sapiens GN=PFKFB3 PE=1 SV=1 - [F2Z2I2_HUMAN] | 3.08 | 2.86 |
| Q5T8R3 | Monocarboxylate transporter 1 (Fragment) OS=Homo sapiens GN=SLC16A1 PE=1 SV=1 - [Q5T8R3_HUMAN] | 3.08 | 4.05 |
| Q5D862 | Filaggrin-2 OS=Homo sapiens GN=FLG2 PE=1 SV=1 - [FILA2_HUMAN] | 3.05 | 0.50 |
| Q68DE0 | Putative uncharacterized protein DKFZp781D2217 OS=Homo sapiens GN=DKFZp781D2217 PE=2 | 3.04 | 4.70 |

| | | | |
|---|---|---|---|
| | SV=1 - [Q68DE0_HUMAN] | | |
| H0Y4T6 | Peptidyl-prolyl cis-trans isomerase NIMA-interacting 4 (Fragment) OS=Homo sapiens GN=PIN4 PE=1 SV=1 - [H0Y4T6_HUMAN] | 3.04 | 13.19 |
| Q7Z5Y0 | EIF4B protein (Fragment) OS=Homo sapiens GN=EIF4B PE=2 SV=1 - [Q7Z5Y0_HUMAN] | 3.02 | 4.58 |
| G3XAN0 | 40S ribosomal protein S20 OS=Homo sapiens GN=RPS20 PE=1 SV=1 - [G3XAN0_HUMAN] | 3.01 | 18.75 |
| H0YEP8 | Serpin H1 (Fragment) OS=Homo sapiens GN=SERPINH1 PE=1 SV=1 - [H0YEP8_HUMAN] | 3.01 | 21.43 |
| Q75L23 | Putative uncharacterized protein PSMC2 (Fragment) OS=Homo sapiens GN=PSMC2 PE=3 SV=1 - [Q75L23_HUMAN] | 3.00 | 3.18 |
| Q9NV86 | cDNA FLJ10873 fis, clone NT2RP4001730, weakly similar to UDP-GLUCOSE:GLYCOPROTEIN GLUCOSYLTRANSFERASE (EC 2.4.1.-) (Fragment) OS=Homo sapiens PE=2 SV=1 - [Q9NV86_HUMAN] | 2.97 | 1.10 |
| D3DWL0 | Plectin 1, intermediate filament binding protein 500kDa, isoform CRA_b OS=Homo sapiens GN=PLEC1 PE=4 SV=1 - [D3DWL0_HUMAN] | 2.96 | 0.76 |
| H7C1I9 | Microtubule-associated serine/threonine-protein kinase 4 (Fragment) OS=Homo sapiens GN=MAST4 PE=1 SV=1 - [H7C1I9_HUMAN] | 2.96 | 0.95 |
| A0A0A0MSD6 | Teneurin-3 OS=Homo sapiens GN=TENM3 PE=1 SV=1 - [A0A0A0MSD6_HUMAN] | 2.95 | 0.83 |
| Q9BTC0 | Death-inducer obliterator 1 OS=Homo sapiens GN=DIDO1 PE=1 SV=5 - [DIDO1_HUMAN] | 2.94 | 0.76 |

| | | | |
|---|---|---|---|
| E5RJH3 | 60S ribosomal protein L30 OS=Homo sapiens GN=RPL30 PE=1 SV=1 - [E5RJH3_HUMAN] | 2.93 | 21.43 |
| Q15365 | Poly(rC)-binding protein 1 OS=Homo sapiens GN=PCBP1 PE=1 SV=2 - [PCBP1_HUMAN] | 2.92 | 3.65 |
| H0YD14 | Myoferlin (Fragment) OS=Homo sapiens GN=MYOF PE=1 SV=1 - [H0YD14_HUMAN] | 2.91 | 0.85 |
| P62861 | 40S ribosomal protein S30 OS=Homo sapiens GN=FAU PE=1 SV=1 - [RS30_HUMAN] | 2.88 | 18.64 |
| Q6ZQN2 | cDNA FLJ46846 fis, clone UTERU3004635, moderately similar to Neuroblast differentiation associated protein AHNAK (Fragment) OS=Homo sapiens PE=2 SV=1 - [Q6ZQN2_HUMAN] | 2.85 | 0.82 |
| B3KU66 | cDNA FLJ39263 fis, clone OCBBF2009571, highly similar to ATP-dependent RNA helicase A (EC 3.6.1.-) OS=Homo sapiens PE=2 SV=1 - [B3KU66_HUMAN] | 2.85 | 2.91 |
| Q9BSE8 | ZNF607 protein OS=Homo sapiens GN=ZNF607 PE=2 SV=1 - [Q9BSE8_HUMAN] | 2.83 | 6.41 |
| Q9H369 | PRO1633 OS=Homo sapiens PE=2 SV=1 - [Q9H369_HUMAN] | 2.83 | 6.56 |
| A8MV73 | Activating transcription factor 7-interacting protein 1 (Fragment) OS=Homo sapiens GN=ATF7IP PE=1 SV=2 - [A8MV73_HUMAN] | 2.82 | 5.24 |
| B4DW08 | cDNA FLJ50886, highly similar to Aconitate hydratase, mitochondrial(EC 4.2.1.3) OS=Homo sapiens PE=2 SV=1 - [B4DW08_HUMAN] | 2.82 | 2.65 |
| Q7Z6E9 | E3 ubiquitin-protein ligase RBBP6 OS=Homo sapiens GN=RBBP6 PE=1 SV=1 - [RBBP6_HUMAN] | 2.82 | 0.84 |

| | | | |
|---|---|---|---|
| P81605 | Dermcidin OS=Homo sapiens GN=DCD PE=1 SV=2 - [DCD_HUMAN] | 2.80 | 10.00 |
| M0QXI6 | RuvB-like 2 (Fragment) OS=Homo sapiens GN=RUVBL2 PE=1 SV=1 - [M0QXI6_HUMAN] | 2.80 | 11.01 |
| Q5T4L4 | 40S ribosomal protein S27 OS=Homo sapiens GN=RPS27 PE=1 SV=1 - [Q5T4L4_HUMAN] | 2.80 | 19.70 |
| A0A024R9T5 | HCG20693, isoform CRA_a OS=Homo sapiens GN=hCG_20693 PE=3 SV=1 - [A0A024R9T5_HUMAN] | 2.78 | 9.46 |
| B7Z1C9 | Chaperonin containing TCP1, subunit 7 (Eta), isoform CRA_a OS=Homo sapiens GN=CCT7 PE=2 SV=1 - [B7Z1C9_HUMAN] | 2.77 | 3.86 |
| Q6ZU52 | Uncharacterized protein KIAA0408 OS=Homo sapiens GN=KIAA0408 PE=1 SV=1 - [K0408_HUMAN] | 2.75 | 2.59 |
| B5LY67 | Uridine monophosphate synthetase isoform E OS=Homo sapiens GN=UMPS PE=2 SV=1 - [B5LY67_HUMAN] | 2.75 | 2.92 |
| G3V4V5 | E3 ubiquitin-protein ligase HECTD1 (Fragment) OS=Homo sapiens GN=HECTD1 PE=1 SV=1 - [G3V4V5_HUMAN] | 2.75 | 1.62 |
